# Superior MAP–EtCO_2_ Target Attainment with Rocuronium-Facilitated Controlled Ventilation in Geriatric Small Dogs: A Retrospective Cohort Study

**DOI:** 10.3390/ani16203203

**Published:** 2026-10-12

**Authors:** Seung-Hyun Kim, Eun-Ji Baek, Chun-Sik Bae

**Affiliations:** 1 KSH Surgical Animal Medical Center, Gwangju 61617, Republic of Korea; 100dmswl1@gmail.com; 2 Department of Veterinary Surgery, College of Veterinary Medicine, Chonnam National University, Gwangju 61186, Republic of Korea

**Keywords:** geriatric dogs, rocuronium, sugammadex, controlled ventilation, quantitative neuromuscular monitoring, mean arterial pressure, end-tidal carbon dioxide

## Abstract

Comparative evidence for integrated ventilation–neuromuscular blockade–reversal strategies is limited in geriatric small dogs. This single-center retrospective cohort included 288 dogs aged ≥12 years and weighing ≤7 kg undergoing orthopedic surgery or thoracolumbar hemilaminectomy. Initially planned strategies were spontaneous ventilation without neuromuscular blockade (A), controlled ventilation without blockade (B), rocuronium–controlled ventilation–sugammadex (C), vecuronium–controlled ventilation–sugammadex (D), or vecuronium–controlled ventilation–neostigmine–atropine (E). The primary outcome was the percentage of the entire primary physiologic window with concurrent mean arterial pressure ≥ 60 mmHg and end-tidal carbon dioxide 35–55 mmHg. Adjusted means were A 58.9%, B 59.3%, C 86.6%, D 74.0%, and E 67.8%. Group C exceeded A, B, and D by 27.7 (95% confidence interval, 26.3–29.2), 27.3 (25.9–28.8), and 12.7 (11.2–14.1) percentage points; B and A differed by 0.4 points (−0.8–1.6). Ventilator asynchrony occurred in 3.4% of C versus 73.2% of B. Adjusted mean times to normalized train-of-four ratio ≥ 0.90 were 1.41, 2.31, and 7.43 min in Groups C–E, respectively. Observed early respiratory and hard 48 h event risks were 3.4% and 1.7% in C, 40.7% and 18.6% in A, and 44.6% and 17.9% in B. The rocuronium-facilitated strategy was associated with superior concurrent target attainment; nonrandomized strategy selection precludes causal safety conclusions.

## Simple Summary

Maintaining blood pressure and ventilation during anesthesia can be difficult in older small dogs, particularly when surgery requires immobility. We reviewed 288 dogs aged at least 12 years and weighing no more than 7 kg that underwent orthopedic or spinal surgery under one of five anesthetic strategies. Dogs managed with rocuronium, controlled ventilation, quantitative neuromuscular monitoring, and sugammadex reversal met the concurrent blood-pressure and exhaled-carbon-dioxide targets for about 87% of the assessed period, versus about 59% in dogs initially managed with spontaneous ventilation or controlled ventilation without a muscle relaxant. Compared with controlled ventilation without a muscle relaxant, this strategy was associated with less ventilator mismatch; compared with the vecuronium–sugammadex strategy, it was associated with lower inhaled-anesthetic and fentanyl-infusion exposures and faster muscle recovery. After vecuronium, sugammadex was also associated with faster recovery than neostigmine–atropine; the rocuronium-based strategy had the lowest observed rates of early breathing problems and prespecified serious events within 48 h. Because treatment was selected clinically rather than randomly, this study cannot prove that this strategy caused the better outcomes. Nevertheless, the findings support prospective evaluation of this coordinated approach for older small dogs requiring controlled ventilation and surgical immobility.

## 1. Introduction

General anesthesia in geriatric dogs requires simultaneous management of ventilation, circulation, anesthetic depth, surgical immobility, and recovery. Large veterinary datasets have associated anesthesia-related mortality with a poorer preanesthetic physical status and other patient- and procedure-related factors, including advanced age and urgent care [1,2,3,4]. A large international canine cohort reported an anesthesia-related mortality of 0.69% from premedication to 48 h after extubation [4]. Mortality alone, however, does not capture nonfatal hemodynamic and ventilatory disturbances during anesthesia. Mean arterial pressure (MAP) and end-tidal carbon dioxide tension (EtCO_2_) are routinely used to guide intraoperative cardiovascular and ventilatory management, and a MAP threshold of approximately 60 mmHg is commonly applied in anesthetized dogs [5,6,7]. Evidence from human noncardiac surgery further indicates that the depth and duration of hypotension convey information beyond a single binary measurement, although associated outcome thresholds cannot be transferred directly to dogs [8,9]. Evaluating the duration for which adequate ventilation and arterial pressure are achieved concurrently may therefore provide a more integrated description of primary-window physiologic control than either variable considered alone.

The ventilatory strategy is one of several interacting determinants of arterial pressure during general anesthesia. Other potential contributors to hypotension include hypovolemia associated with reduced intake or fluid losses, clinically significant cardiac disease including myxomatous mitral valve disease, and renal or endocrine disorders affecting volume regulation. Inhalant anesthetics, including isoflurane and sevoflurane, can cause dose-dependent vasodilation and cardiovascular depression, as well as depress spontaneous ventilation [6]. Positive-pressure ventilation can improve carbon dioxide control, but increased intrathoracic pressure may reduce the pressure gradient for systemic venous return, decreasing right ventricular preload and subsequently limiting left ventricular filling and cardiac output [6,10]. The magnitude of these effects depends on the circulating volume, cardiac function, airway pressures, and lung and chest-wall mechanics; excessive inspiratory pressures or positive end-expiratory pressure may exacerbate hemodynamic compromise [6,10,11]. When respiratory-system compliance is reduced, a greater pressure difference is required to deliver a given tidal volume, although the peak airway pressure also reflects airway resistance [12]. These interactions emphasize the importance of individualizing ventilator settings according to respiratory mechanics, gas exchange, and cardiovascular tolerance. Studies in anesthetized dogs have demonstrated that spontaneous ventilation, positive-pressure ventilation, pressure- and volume-controlled modes, positive end-expiratory pressure, and tidal-volume selection produce different cardiopulmonary profiles [10,11,13]. Nevertheless, initiating controlled ventilation does not necessarily ensure passive, synchronous ventilation when spontaneous respiratory activity persists. Patient–ventilator discordance may prompt repeated setting changes, deeper anesthesia, rescue manual ventilation, or conversion of the planned technique. Neuromuscular blocking agents (NMBAs) can facilitate surgical immobility and controlled ventilation by suppressing skeletal-muscle activity, but they provide neither hypnosis nor analgesia and remove movement as a clinical response to inadequate anesthetic depth or antinociception [14]. An NMBA-containing technique must therefore be considered as a coordinated anesthetic strategy rather than as an isolated drug intervention.

Rocuronium and vecuronium are aminosteroidal NMBAs for which canine clinical and pharmacodynamic experience has been reported. Rocuronium has been administered to dogs as repeated boluses and continuous infusions, and sugammadex can reverse profound blockade produced by either rocuronium or vecuronium [15,16,17]. By contrast, reversal with neostigmine depends on sufficient spontaneous recovery and requires concomitant antimuscarinic treatment [14,18]. Restoration of spontaneous ventilation does not exclude residual neuromuscular blockade in dogs, making objective train-of-four (TOF) assessment important when blockade and reversal are used [19]. Subsequent canine experimental and clinical studies have documented the clinical feasibility of quantitative monitoring and sugammadex reversal and have demonstrated faster recovery from vecuronium-induced blockade with sugammadex than with neostigmine [20,21,22,23]. At the same time, recovery estimates can differ among monitoring technologies, and human recovery thresholds and pulmonary-outcome evidence cannot be extrapolated uncritically to dogs [24,25,26,27].

Most available canine evidence addresses an individual ventilation mode, NMBA, monitoring technology, or reversal agent under experimental conditions or in relatively small clinical series. Routine peri-anesthetic care, however, implements these elements as linked strategies: the planned ventilation mode influences spontaneous respiratory activity; the choice and depth of neuromuscular blockade affect ventilator interaction and immobility; isoflurane and opioid titration occur in that context; and reversal and extubation occur later under quantitative monitoring. To our knowledge, no previously published large-scale clinical cohort of client-owned dogs has jointly evaluated controlled ventilation, aminosteroidal neuromuscular blockade, quantitative neuromuscular monitoring and recovery, pharmacologic reversal, minute-level MAP–EtCO_2_ control, emergence, and early postoperative outcomes within a single comparative framework. This distinction is important because evidence supporting rapid pharmacologic reversal does not itself demonstrate better physiology during the prespecified primary window or improved postoperative clinical safety.

The objective of this retrospective cohort study was therefore to compare five initially planned ventilation–NMBA–reversal strategies in dogs aged at least 12 years and weighing no more than 7 kg undergoing pelvic-limb orthopedic surgery or thoracolumbar hemilaminectomy. The primary outcome was the prespecified, study-derived percentage of the entire primary physiologic window during which MAP and EtCO_2_ simultaneously met their analytic targets. Secondary objectives were to characterize component target-time and dysregulation-burden measures, the time-weighted mean end-tidal isoflurane concentration and fentanyl continuous-infusion rate, ventilatory implementation and asynchrony, quantitative TOF recovery and emergence, hemodynamic support, and pre-extubation and postoperative safety events. The joint target-time measure was intended as an integrated physiologic endpoint, not as a validated morbidity scale or a substitute for its individual cardiovascular and ventilatory components.

Five clinically motivated contrasts were prespecified. We hypothesized that rocuronium-facilitated controlled ventilation would be associated with greater concurrent MAP–EtCO_2_ target attainment than spontaneous or controlled ventilation without an NMBA, and with more favorable primary-window physiology and quantitative neuromuscular recovery than vecuronium-facilitated controlled ventilation when both strategies included planned sugammadex reversal. We further hypothesized that controlled ventilation without an NMBA would provide less consistent benefit over spontaneous ventilation, and that sugammadex would be associated with faster quantitative neuromuscular recovery and emergence than neostigmine–atropine after vecuronium. These comparisons were intended to evaluate associations between implemented anesthetic strategies. Comparisons involving NMBA choice and safety outcomes were interpreted as strategy-level associations rather than causal effects of isolated drugs.

## 2. Materials and Methods

### 2.1. Study Design, Setting, Ethics, and Reporting

This single-center retrospective cohort study included client-owned dogs that underwent pelvic-limb orthopedic surgery or thoracolumbar hemilaminectomy for intervertebral disc extrusion at the KSH Surgical Animal Medical Center, Gwangju, Republic of Korea, between January 2025 and May 2026. All clinical exposures, physiologic measurements, and postoperative outcomes had been recorded during routine clinical care before commencement of the retrospective review. No anesthetic, ventilatory, neuromuscular-blocking, or reversal intervention was assigned for research purposes. Because this study was limited to the secondary analysis of existing clinical records and involved no research-mandated intervention or deviation from standard clinical care, formal review and approval by an Institutional Animal Care and Use Committee (IACUC) were not required under the institutional guidelines of the KSH Surgical Animal Medical Center.

The surgical and anesthetic framework, monitoring equipment, physiologic treatment targets, and perioperative decision algorithms remained unchanged throughout the study period. The five ventilation–neuromuscular blockade–reversal strategies represented clinical exposure groups rather than successive protocol eras. The same operating surgeon and the same two designated anesthetists provided surgical and peri-anesthetic care throughout the study period. Written informed owner consent was obtained for all surgical and anesthetic procedures and for the research use and publication of de-identified clinical data. This study was reported in accordance with the STROBE-Vet statement for observational veterinary studies [28].

### 2.2. Eligibility Criteria and Case Identification

Electronic medical, surgical, anesthesia, medication-administration, and perioperative monitoring records were screened for procedures performed during the study period. Of 1141 surgical records screened, 288 fulfilled all eligibility criteria and were included.

Dogs were eligible if they were at least 12 years old and weighed no more than 7 kg; underwent either unilateral or bilateral pelvic-limb orthopedic surgery—including fracture repair, femoral head and neck ostectomy (FHNO), tibial plateau leveling osteotomy (TPLO), or surgical correction of patellar luxation—or thoracolumbar hemilaminectomy for intervertebral disc extrusion; had identifiable anesthesia-start and anesthesia-end timestamps; had sufficient valid mean arterial pressure (MAP) and end-tidal carbon dioxide tension (EtCO_2_) data to construct the primary analysis window, with a paired MAP–EtCO_2_ coverage of at least 70%; could be classified unambiguously into one of the five prespecified strategies; and had the perioperative medication, ventilation, recovery, and safety records required for review. For dogs that received a neuromuscular blocking agent (NMBA), interpretable neuromuscular monitoring and reversal records were additionally required.

Exclusion criteria were any surgical procedure outside the two prespecified surgical strata, inability to define the primary analysis window, paired MAP–EtCO_2_ coverage < 70%, ambiguous initial strategy assignment, irreconcilable inconsistencies among anesthesia, medication, and monitoring records, and repeat surgery in the same dog. Each dog contributed one anesthetic episode, and all 288 included procedures were performed in different dogs. Age, body weight, sex and neuter status, breed, ASA physical status, cardiac status, renal or respiratory risk, urgency, surgical stratum, and procedural complexity were abstracted from the preanesthetic, surgical, anesthesia, and perioperative records. ASA physical status, cardiac status, renal or respiratory risk, urgency, and procedural complexity were retained as contemporaneously documented in the clinical records and were not retrospectively reclassified for study purposes. Category labels and allowed values are provided in Supplementary Table S1.

### 2.3. Standardized Surgical and Anesthetic Management

Food was withheld for 8 h before anesthesia. Other preanesthetic preparation was performed according to the institutional protocol. After placement of an intravenous catheter, remifentanil 1 µg kg^−1^ was administered intravenously over 1 min as a pre-induction opioid analgesic–sedative adjunct. General anesthesia was then induced with alfaxalone 2 mg kg^−1^ administered as a fixed intravenous bolus.

After induction, the trachea was intubated with a cuffed endotracheal tube. Tube internal diameter was selected according to patient size and airway dimensions using the same institutional airway-sizing protocol. Correct tracheal placement and an adequate airway seal were confirmed clinically and by capnography.

Anesthesia was maintained with isoflurane delivered in oxygen through the integrated circle rebreathing system of an A5-V veterinary anesthesia workstation (Shenzhen Comen Medical Instruments Co., Ltd., Shenzhen, China). Physiologic monitoring was performed using a separate Brio X7 multiparameter patient monitor (Bionet Co., Ltd., Seoul, Republic of Korea), as detailed in Section 2.6. Oxygen was used as the sole carrier gas, and fresh-gas flow was adjusted according to body weight and circuit requirements. Inspired oxygen concentration was continuously monitored using the oxygen analyzer integrated into the A5-V anesthesia workstation. End-tidal isoflurane concentration was adjusted using a consistent clinically guided approach based on anesthetic depth, cardiovascular and respiratory responses to surgical stimulation, blood pressure, ventilation, and individual comorbidity. The concentration was increased when clinically interpretable signs indicated inadequate anesthetic depth and decreased when excessive depth or cardiovascular depression was suspected in the absence of an ongoing nociceptive response; no single fixed numerical titration threshold was used.

Fentanyl was used for intraoperative analgesia in all dogs. An intravenous loading or rescue bolus of 2–5 µg kg^−1^ was followed by a continuous rate infusion initiated at 5 µg kg^−1^ h^−1^ and titrated within a range of 2–8 µg kg^−1^ h^−1^ using a consistent clinically guided approach. Upward titration or a rescue bolus was prompted by sustained autonomic responses temporally associated with surgical stimulation or other interpretable signs of inadequate antinociception; downward titration was undertaken when cardiovascular depression or excessive anesthetic depth was suspected without an ongoing nociceptive response. In NMBA-treated dogs, movement was not used as an indicator of antinociceptive adequacy. The time-weighted mean fentanyl CRI rate was calculated as the cumulative infusion-rate–time integral divided by the entire primary-window duration; intervals during which the infusion was not running were assigned a rate of zero. Supplemental fentanyl bolus doses were excluded from this calculation. The time-weighted mean end-tidal isoflurane concentration was calculated as the cumulative EtISO–time integral divided by the entire primary-window duration; intervals before isoflurane administration were assigned a concentration of zero. No additional intraoperative systemic analgesic infusion or locoregional analgesic technique was used.

Lactated Ringer’s solution (inno.N Hartmann Sol.; HK inno.N Corp., Seoul, Republic of Korea), a balanced isotonic crystalloid, was administered during anesthesia. The maintenance rate and subsequent adjustments were determined by the attending anesthetist according to body weight, cardiac and renal status, blood pressure, estimated fluid loss, and clinical perfusion. When MAP remained <60 mmHg after confirmation of the NIBP measurement and reassessment of anesthetic depth, heart rate and rhythm, esophageal temperature, and estimated circulating volume, or when other clinical findings suggested hypovolemia or impaired tissue perfusion, a crystalloid fluid challenge of 5 mL kg^−1^ was administered intravenously over approximately 10 min. MAP and clinical perfusion variables, including heart rate, pulse quality, mucous-membrane color, capillary refill time, and SpO_2_, were reassessed after each challenge. Further fluid challenges were administered only when the initial response and reassessment of volume status supported additional fluid administration; repeat administration was reduced or withheld in dogs with cardiac or renal disease or evidence of fluid overload. A qualifying fluid challenge was recorded separately from routine maintenance-fluid administration.

Hypotension was managed using a stepwise, clinically guided approach. When MAP remained <60 mmHg after confirmation of the NIBP reading and reassessment of anesthetic depth, end-tidal isoflurane concentration, heart rate and rhythm, esophageal temperature, ventilation, and estimated circulating volume, the vaporizer setting was reduced when clinically permissible, and reversible contributing factors were corrected. Dopamine was initiated at 5 µg kg^−1^ min^−1^ for persistent hypotension considered attributable to inadequate cardiac contractility or vasodilation and was titrated to the lowest effective rate according to serial MAP, heart rate, and continuous esophageal ECG responses, with the objective of maintaining MAP ≥ 60 mmHg and adequate clinical perfusion. The infusion rate did not exceed 15 µg kg^−1^ min^−1^.

Active thermal management was provided with a forced-air warming blanket throughout anesthesia and was adjusted according to continuously measured esophageal temperature. Although isoflurane concentration, fentanyl infusion rate, ventilator settings, fluids, and cardiovascular support were titrated to individual clinical requirements, the core anesthetic protocol and treatment algorithms did not change during the study period.

### 2.4. Definition of the Five Anesthetic Strategies

Dogs were classified according to the initially planned ventilation–NMBA–reversal strategy. Group A comprised dogs managed with conventional spontaneous ventilation without an NMBA. Group B comprised dogs assigned to planned controlled ventilation without an NMBA. Group C comprised dogs treated with rocuronium, controlled ventilation, and sugammadex reversal. Group D comprised dogs treated with vecuronium, controlled ventilation, and sugammadex reversal. Group E comprised dogs treated with vecuronium, controlled ventilation, and neostigmine–atropine reversal.

The initial strategy was selected for clinical care by the attending anesthetist before implementation of controlled ventilation or neuromuscular blockade. Selection considered the anticipated need for surgical immobility and muscle relaxation, expected procedural duration, respiratory pattern, cardiopulmonary status, and individual clinical judgment. No strategy was assigned for research purposes, and no formal fixed allocation algorithm was used.

The initial strategy documented before implementation of ventilation or neuromuscular blockade determined group classification and was cross-checked against the ventilator record and medication-administration record. Brief rescue manual ventilation, transient spontaneous respiratory activity, and an unplanned conversion undertaken for patient safety did not alter the initial classification. Such deviations were retained as observed measures of ventilation exposure, ventilator-setting adjustment, and unplanned ventilatory intervention.

A major crossover was defined as a deviation that materially replaced a core component of the initially assigned strategy: controlled ventilation for more than 20% of the post-intubation ventilatory-assessment window in a dog assigned to spontaneous ventilation; controlled ventilation for less than 80% of the post-intubation ventilatory-assessment window in a dog assigned to a controlled-ventilation strategy; unplanned administration of an NMBA to a no-NMBA group; administration of an NMBA inconsistent with the assigned bundle; or use of a reversal agent inconsistent with the assigned bundle. Brief rescue ventilation that accounted for no more than 20% of the post-intubation ventilatory-assessment window and ventilator-setting changes that did not alter the assigned strategy were not considered major crossovers. Dogs with a major crossover retained their initially assigned group in the primary intention-to-strategy analysis and were excluded from the per-strategy sensitivity analysis.

### 2.5. Ventilatory Management

All mechanically controlled ventilation was delivered in pressure-regulated volume-controlled (PRVC) mode using the A5-V veterinary anesthesia workstation. This included planned controlled ventilation in Groups B–E and unplanned conversion from spontaneous ventilation in Group A. Initial settings comprised a target tidal volume of 10–12 mL kg^−1^ of actual body weight, a respiratory rate of 10–15 breaths min^−1^, positive end-expiratory pressure of 3–4 cmH_2_O, and an inspiratory or high-pressure limit not exceeding 30 cmH_2_O. The selected tidal-volume range has previously been evaluated in anesthetized dogs [13].

Respiratory rate was adjusted first to alter minute ventilation. Target tidal volume, positive end-expiratory pressure, inspiratory time or inspiratory-to-expiratory ratio, and the pressure limit were subsequently adjusted in response to lung compliance, peak airway pressure, capnographic waveform, oxygen saturation, hemodynamic response, or patient–ventilator interaction. The clinical objective was to maintain EtCO_2_ between 35 and 50 mmHg whenever feasible. This clinical management range was narrower than the prespecified analytic target range of 35–55 mmHg. The initial ventilator settings and pressure limit described above represent prescribed targets and limits rather than patient-level measurements or time-averaged summaries of delivered ventilation.

Ventilator asynchrony was assessed only in dogs assigned to planned controlled ventilation (Groups B–E); unplanned conversion episodes in Group A were not included in this variable. Within Groups B–E, ventilator asynchrony was defined as documented patient–ventilator discordance identified from respiratory effort, airway-pressure or flow waveforms, capnography, or clinical signs. Qualifying patterns included ineffective triggering, double triggering, breath stacking, active expiration against an inspiratory breath, bucking, or repeated out-of-phase spontaneous effort that prompted adjustment of anesthesia or ventilator settings. Isolated spontaneous respiratory effort without documented discordance or intervention was not classified as asynchrony.

Manual ventilation was defined as hand-delivered positive-pressure breaths using the reservoir bag for no more than 5 consecutive min, without activation of the mechanical ventilator or a sustained change in the planned ventilation mode. In dogs initially managed with spontaneous ventilation, conversion to controlled ventilation was defined as an unplanned transition to mechanical controlled ventilation lasting more than 5 consecutive min. Hand-delivered breaths were not included in the calculation of controlled-ventilation exposure. For quantification of ventilation-mode exposure, the ventilatory-assessment window began at tracheal intubation and ended at discontinuation of isoflurane. This window was distinct from the primary physiologic window used for the joint target-time outcome.

The controlled-ventilation percentage was calculated as follows:

$$\mathrm{Controlled ventilation percentage}=100\times \frac{\mathrm{controlled mechanical}-\mathrm{ventilation duration}}{\mathrm{post}-\mathrm{intubation ventilatory}-\mathrm{assessment}-\mathrm{window duration}}$$

A nonzero controlled-ventilation percentage in group A therefore represented an unplanned period of mechanical controlled ventilation rather than brief hand-delivered breaths.

A ventilator-setting change was counted whenever the ventilation mode, target tidal volume, respiratory rate, positive end-expiratory pressure, pressure limit, inspiratory time, inspiratory-to-expiratory ratio, or trigger setting was altered after initial setup. Simultaneous modification of multiple parameters at the same documented time was counted as one setting-change event. Initiation of a manual-ventilation episode was counted once as a ventilation-mode change, irrespective of the number of hand-delivered breaths. Initial connection and configuration of the ventilator were not counted.

### 2.6. Physiologic Data Acquisition and Signal Processing

Physiologic monitoring was performed using a separate Brio X7 multiparameter patient monitor (Bionet Co., Ltd., Seoul, Republic of Korea). Immediately after tracheal intubation, a combined esophageal electrocardiography and temperature probe was positioned to obtain a stable electrocardiographic tracing. Thereafter, esophageal ECG, esophageal temperature, pulse oximetry, capnography, and respiratory waveforms were displayed and observed continuously. EtCO_2_ was measured using mainstream capnography, with the sensor positioned at the airway between the endotracheal-tube connector and the breathing circuit. A pediatric low-dead-space capnography airway adapter was used, without additional filters, elbow adapters, or extension tubing at the patient connection, to minimize additional apparatus dead space. The pulse-oximetry probe was placed on the tongue, and measurements were accepted only when accompanied by a stable plethysmographic waveform. MAP was measured noninvasively by oscillometry at automatic 1 min intervals. A cuff with a width approximating 40% of the measurement-site circumference was placed around an available pelvic limb or the base of the tail according to surgical access and patient positioning. The selected cuff site was maintained throughout the primary window.

The primary window began at the recorded time of alfaxalone administration and ended at the recorded time at which isoflurane administration was discontinued. In the routine clinical workflow for this cohort, tracheal intubation was typically completed within approximately 5–10 s after the intravenous alfaxalone bolus. This brief interval fell within the first induction-anchored 1 min analytic epoch and was not separately resolved in the available minute-level records.

Capnographic data were considered valid when a recognizable respiratory waveform with an expiratory plateau and consistent breath detection was present. Measurements associated with capnography-sensor disconnection or malposition, contamination or obstruction of the airway adapter, major circuit leakage, an absent expiratory plateau, flushing, calibration failure, movement artifact, or documented equipment malfunction were considered invalid. An oscillometric MAP measurement was considered valid when the monitor reported a successful measurement cycle, cuff size and placement remained appropriate, and no error code or clinically evident motion artifact was present. Failed or implausible cycles were not treated as observed measurements.

Physiologic data were aligned to uniform 1 min analytic epochs. Within each epoch, EtCO_2_ was represented by the median of all valid observations, and MAP was assigned using the most recent valid oscillometric measurement. An MAP value was carried forward for no longer than 2 min; epochs occurring more than 2 min after the last valid MAP measurement were considered MAP-invalid. An isolated EtCO_2_ gap of no more than 2 min bounded by valid observations was linearly interpolated; longer gaps and unbounded gaps remained EtCO_2_-invalid. No extrapolation across a primary-window boundary was performed. An epoch was paired-valid only when both EtCO_2_ and MAP were valid. Analytic epochs were anchored at the recorded time of alfaxalone administration and continued in consecutive 1 min intervals until discontinuation of isoflurane. The final partial epoch was retained using its actual duration. Validity was assigned at the epoch level rather than on a second-by-second basis. An epoch was classified as paired-valid when it contained a valid EtCO_2_ summary and a valid MAP assignment according to the prespecified signal-processing rules, even when valid observations did not span the entire epoch. Accordingly, a pre-intubation interval was classified as missing only when it resulted in an epoch containing no valid EtCO_2_ observation. Paired-coverage and target-time percentages were duration-weighted using the classified epochs and rounded to one decimal place.

An epoch was paired-valid only when both EtCO_2_ and MAP were valid. Paired coverage was calculated as follows:

$$\mathrm{Paired coverage}=100\times \frac{\mathrm{cumulative duration of paired}-\mathrm{valid epochs}}{\mathrm{entire primary}-\mathrm{window duration}}.$$

Paired coverage was calculated as 100 multiplied by the cumulative duration of paired-valid epochs divided by the entire primary-window duration. An epoch was classified as paired-valid when both EtCO_2_ and MAP were valid according to the prespecified signal-processing rules.

Signal gaps remained identifiable and were not replaced with group-level, patient-level, or adjacent-case values.

### 2.7. Primary Outcome Definition

The primary physiologic outcome was the joint target-time percentage: the percentage of the entire primary window during which MAP and EtCO_2_ simultaneously met the prespecified targets. MAP was within target at ≥60 mmHg, and EtCO_2_ was within target at 35–55 mmHg.

For a primary window of duration T, divided into epochs j of duration Δt_j_, an indicator I_j_ was assigned a value of 1 when epoch j was paired-valid and both physiologic targets were met, and 0 otherwise. The joint target-time percentage was calculated as

$$\mathrm{Joint target}-\mathrm{time percentage}=100\times \frac{\Sigma_{j}{\Delta t}_{j}I_{j}}{T}.$$

The denominator was the entire primary window rather than only the paired-valid monitoring time. An epoch with missing or invalid MAP or EtCO_2_ remained in the denominator but did not contribute to the numerator.

The individual MAP and EtCO_2_ target-time percentages were calculated analogously, with time in the respective target divided by the entire primary-window duration. Consequently, the joint target-time percentage could not exceed paired coverage, the MAP target-time percentage, or the EtCO_2_ target-time percentage.

### 2.8. Physiologic Burden Metrics

The EtCO_2_ dysregulation burden and hypotension burden were prespecified supportive outcomes that incorporated both the magnitude and duration of physiologic deviation. They were study-derived measures and were not treated as validated veterinary clinical scores.

For each valid MAP epoch, hypotension depth (H_j_) was defined as

*H_j_* = max(60 − MAP*_j_*, 0).

Hypotension burden was then calculated as

Hypotension burden =ΣjHjΔtjvalid MAP monitoring time h.

For each valid EtCO_2_ epoch, dysregulation depth (D_j_) was defined as

*D_j_* = max(35 − EtCO_2,*j*_, 0) + max(EtCO_2,*j*_ − 55, 0).

EtCO_2_ dysregulation burden was then calculated as

$${\mathrm{EtCO}}_{2}\;\mathrm{dysregulation}\;\mathrm{burden}=\frac{\Sigma_{j}D_{j}{\Delta t}_{j}}{\mathrm{valid}\;{\mathrm{EtCO}}_{2}\;\mathrm{monitoring}\;\mathrm{time}\;(h)}.$$

Both burdens were expressed as mmHg·min per valid monitoring hour. Only valid observations contributed to the corresponding integral and denominator, and the same rectangular numerical-summation procedure was applied to all groups. The MAP threshold of 60 mmHg was selected as a consensus-aligned study threshold rather than an absolute biologic boundary. A survey of veterinary anesthesiology specialists reported mean MAP thresholds of approximately 62 mmHg for defining intraoperative hypotension and approximately 61 mmHg for initiating treatment [5], and the selected threshold was also consistent with the 2020 AAHA Anesthesia and Monitoring Guidelines for Dogs and Cats [6].

### 2.9. Quantitative Neuromuscular Monitoring

Quantitative neuromuscular monitoring was performed in groups C, D, and E using a Stimpod NMS450X three-axis acceleromyograph (Xavant Technology, Pretoria, South Africa). Train-of-four (TOF) monitoring was not applicable to groups A and B because these dogs did not receive an NMBA.

Stimulating electrodes were placed over the ulnar nerve on the medial aspect of the antebrachium, and the acceleromyography sensor was secured to the dorsal surface of the ipsilateral paw while allowing the monitored paw and digit to move freely. The same limb and sensor configuration were maintained throughout monitoring. The stimulation current was set at 35 mA for all TOF measurements. Four 200 µs electrical stimuli were delivered at 2 Hz, and TOF assessments were repeated at approximately 1 min intervals. Electrode and sensor placement followed the configuration described by Araos and Martin-Flores [22].

Before the initial NMBA dose, repeated artifact-free baseline measurements were obtained after electrode and sensor positioning and stabilization of the signal. The final technically valid TOFR obtained after stable baseline responses had been observed was designated the individual baseline TOFR. Baseline TOFR was calculated as T4/T1. Each subsequent quantitative TOFR was normalized to the individual baseline as follows:

$$\mathrm{Normalized TOFR}=\frac{\mathrm{observed raw TOFR}}{\mathrm{individual baseline TOFR}}.$$

Normalized values greater than 1.00 were retained without truncation.

TOF count (TOFC) and TOFR at reversal were obtained from the final valid assessment within 30 s before reversal-agent administration. When TOFC was 0, TOFR was undefined and treated as missing. When TOFC was 1–3, T4 was absent and TOFR was operationally coded as 0.00; this value was not interpreted as a device-generated quantitative ratio. A quantitative T4/T1 ratio was calculated when TOFC was 4. TOFC was retained separately to characterize the depth of neuromuscular block at reversal and, in Groups C and D, to determine the sugammadex dose.

Time to normalized TOFR ≥ 0.90 was defined as the elapsed time from reversal-agent administration to the first of two consecutive valid normalized TOFR measurements ≥ 0.90. Time to normalized TOFR ≥ 1.00 was defined analogously using the first of two consecutive valid measurements ≥ 1.00. The extubation TOFR was the final valid normalized TOFR obtained within 60 s before extubation. Recovery to normalized TOFR ≥ 0.90 was the principal quantitative neuromuscular-recovery endpoint, whereas recovery to normalized TOFR ≥ 1.00 was a supportive quantitative endpoint.

When normalized TOFR ≥ 1.00 had not been reached before extubation, neuromuscular monitoring continued after extubation at approximately 1 min intervals until two consecutive valid normalized TOFR measurements ≥ 1.00 were obtained. Measurements affected by voluntary movement, accelerometer displacement, loss of electrode contact, or other visible artifact were classified as invalid and repeated after signal stabilization.

### 2.10. Neuromuscular Block Reversal and Extubation

The target initial intravenous doses of rocuronium and vecuronium were 0.6 and 0.1 mg kg^−1^, respectively. Target supplemental doses were rocuronium 0.1–0.2 mg kg^−1^ or vecuronium 0.03–0.04 mg kg^−1^ when TOFC had recovered to ≥2 and continued neuromuscular blockade was required for surgical conditions or ventilator synchrony. Redosing was response-guided rather than performed at fixed intervals. The cumulative NMBA dose was calculated as the sum of all initial and supplemental doses divided by body weight.

In groups C and D, sugammadex 4 mg kg^−1^ IV was administered when the TOFC immediately before reversal was 0 or 1, and sugammadex 2 mg kg^−1^ IV was administered when TOFC was 2–4. In Group E, neostigmine was administered only after spontaneous recovery to a TOFC of at least 2. In group E, vecuronium-induced neuromuscular block was reversed with neostigmine at approximately 0.04 mg kg^−1^ IV; actual administered doses ranged from 0.033 to 0.046 mg kg^−1^. Neostigmine was coadministered with atropine 0.02 mg kg^−1^ IV. The planned atropine dose was part of the group E reversal strategy and was not classified as rescue anticholinergic treatment. Any additional atropine or glycopyrrolate administered for clinically significant bradyarrhythmia was classified separately as rescue treatment. In Groups C–E, the assigned reversal agent was administered after discontinuation of isoflurane. Oxygen flow during circuit washout was adjusted clinically and was not standardized as a fixed case-level exposure.

The interval from the final NMBA dose to reversal-agent administration was calculated in minutes. Esophageal temperature was obtained from the measurement closest to and within 10 min before reversal-agent administration, and the end-tidal isoflurane concentration was obtained from the measurement closest to and within 5 min before reversal. Reversal-to-extubation time was calculated from reversal-agent administration to removal of the endotracheal tube. Inhalant-off-to-extubation time was calculated from discontinuation of isoflurane to removal of the endotracheal tube.

Extubation was performed after adequate spontaneous ventilation, acceptable oxygenation, a stable cardiovascular status, recovery of protective airway reflexes, purposeful swallowing or appropriate jaw tone, and absence of an immediate need for continued airway control had been confirmed. In NMBA-treated dogs, a normalized TOFR ≥ 0.90 was additionally required before extubation. These extubation criteria were applied using the same clinical checklist by one of the two designated anesthetists throughout the study period.

Postoperative analgesic and sedative management followed the same institutional protocol across all strategy groups and was not determined by the initially planned ventilation–NMBA–reversal strategy.

After extubation, all dogs routinely received supplemental oxygen in an oxygen cage, irrespective of whether a respiratory abnormality was suspected. Oxygen supplementation was discontinued once the respiratory rate had stabilized at ≤40 breaths min^−1^ and no increased respiratory effort, cyanosis, hypoxemia, or other clinically apparent respiratory abnormality was present. Respiratory pattern, airway patency, SpO_2_, heart rate and rhythm, temperature, mentation, and neuromuscular recovery were observed until the predefined recovery criteria were met. Routine oxygen supplementation alone was not classified as a respiratory event. Escalation beyond routine recovery care—including oxygen supplementation that was prolonged, reinitiated, or intensified because of documented hypoxemia, airway obstruction, or clinically abnormal respiratory effort, reintubation, or assisted ventilation—was classified as a postoperative respiratory event.

### 2.11. Safety Outcomes

Intraoperative and postoperative safety outcomes were classified from contemporaneous anesthesia, treatment, and hospitalization records using prespecified study definitions. Pre-extubation safety events were assessed from alfaxalone administration through extubation. This pre-extubation safety window was distinct from the primary physiologic window, which ended at discontinuation of isoflurane. For the prespecified safety variables, the term intraoperative encompassed the entire pre-extubation safety window, including emergence after isoflurane discontinuation. Postoperative time zero was the time of extubation. For ECG- and SpO_2_-dependent safety outcomes, ascertainment began with the first technically reliable signal obtained immediately after tracheal intubation.

A clinically significant intraoperative bradyarrhythmia was defined as bradycardia or another bradyarrhythmia confirmed on continuous esophageal ECG that prompted pharmacologic treatment, interruption or adjustment of anesthetic management, or another active intervention during the pre-extubation safety window. No uniform absolute heart-rate cutoff was used. The attending anesthetist assessed the severity of the rate or rhythm disturbance in relation to the patient’s baseline heart rate, concurrent blood pressure and clinical perfusion findings, and the potential for cardiovascular deterioration when deciding whether intervention was required [6]. Monitor artifacts without clinical confirmation were excluded. Intraoperative hypoxemia was defined as at least two consecutive SpO_2_ measurements < 94% with an adequate plethysmographic waveform or an oxygenation-related intervention prompted by a reliable low SpO_2_ value. Isolated low values with a poor signal quality were excluded, and the hypoxemia status was classified as not assessable when reliable SpO_2_ information was insufficient.

Dopamine use was defined as any dopamine infusion initiated during the pre-extubation safety window to treat persistent hypotension or impaired perfusion; the maximum infusion rate was also recorded. Epinephrine use was defined as any systemic intravenous bolus or infusion administered during the pre-extubation safety window for emergency cardiovascular support; local infiltration was excluded. A fluid-bolus event was recorded when at least one qualifying crystalloid fluid challenge described in Section 2.3 was administered during the pre-extubation safety window; routine maintenance-fluid administration and minor pump-rate adjustments were not counted. Rescue anticholinergic treatment comprised atropine 0.02 mg kg^−1^ IV or glycopyrrolate 0.01 mg kg^−1^ IV administered during the pre-extubation safety window for clinically significant bradycardia or bradyarrhythmia. The attending anesthetist individualized drug selection according to the clinical situation and the expected onset and duration of action. Atropine was preferred when a prompt increase in heart rate was prioritized, whereas glycopyrrolate was preferred when a more sustained vagolytic effect was desired. Scheduled atropine coadministered with neostigmine in Group E was excluded from rescue treatment. An early respiratory event within 0–3 h after extubation comprised a reliable SpO_2_ measurement < 94% requiring intervention, apnea or clinically important hypoventilation, upper-airway obstruction, assisted ventilation, reintubation, or new or escalated oxygen treatment beyond routine recovery care. A major event occurring more than 3 and up to 12 h after extubation comprised clinically important respiratory or cardiovascular deterioration requiring new or sustained escalation of oxygen therapy, assisted ventilation, reintubation, vasopressor or inotropic treatment, emergency antiarrhythmic treatment, cardiopulmonary resuscitation, or unplanned escalation of care. A hard event within 48 h after extubation comprised death or euthanasia, cardiopulmonary arrest or resuscitation, reintubation, prolonged assisted ventilation, or another major cardiopulmonary complication resulting in unplanned intensive-care escalation.

The 0–3 h, >3–12 h, and 48 h windows and their composites were prespecified study-defined outcomes rather than validated veterinary sedation, pain, respiratory, or recovery scales. The windows were not mutually exclusive; an event within 48 h could also meet an early or major-event definition. Routine postoperative oxygen supplementation under the standard recovery protocol, planned postoperative hospitalization, scheduled atropine administered with neostigmine, and treatments unrelated to cardiopulmonary safety were not counted as events. Completeness of follow-up through 48 h was verified from electronic inpatient records, including clinician progress notes, nursing observation charts, and medication and treatment logs.

### 2.12. Data Extraction and Quality Control

Records were screened, and case-level data were extracted using a standardized electronic form and data dictionary finalized before inferential analysis. Source documents included electronic medical, preanesthetic, surgical, anesthesia, medication-administration, ventilator, quantitative neuromuscular-monitoring, physiologic-monitor, and postoperative clinician and nursing records through 48 h after extubation. Variable definitions, units, permissible values, and group-specific applicability are provided in Supplementary Table S1; derived variables and outcomes followed the definitions in Section 2.5, Section 2.6, Section 2.7, Section 2.8, Section 2.9, Section 2.10 and Section 2.11.

Each anesthetic episode received a de-identified case identifier. Original hospital identifiers were used only to identify repeat procedures and confirm that each dog contributed one episode, after which they were excluded from the analysis dataset. Initial strategy assignment was cross-checked against ventilator, NMBA, reversal-agent, and neuromuscular-monitoring records, as specified in Section 2.4. Physiologic data were processed identically across groups using the prespecified signal-validity, epoch-construction, interpolation, carry-forward, and duration-weighting rules in Section 2.6, Section 2.7 and Section 2.8. Apart from those rules, missing case-level values were not imputed.

Quality control included range, permissible-value, group-applicability, temporal-sequence, mathematical-consistency, and cross-variable logical checks. These checks assessed consistency among strategy assignment, ventilation exposure, drug administration, reversal conditions, quantitative TOF recovery, extubation, supportive treatments, and safety-event windows; joint target time was required not to exceed paired coverage or either component target time. Structurally inapplicable values were distinguished from ‘Not documented’ and ‘Not assessable’ in accordance with Supplementary Table S1. Every flagged entry was rechecked against the source records, and cases with unresolved discrepancies meeting the prespecified exclusion criteria were excluded. The analysis dataset was finalized after completion of these checks and before inferential analysis.

### 2.13. Statistical Analysis

The original statistical analysis plan was finalized after data quality control and before examination of between-group inferential results. The individual dog was the unit of analysis, and all eligible cases were analyzed according to the initially planned strategy, as defined in Section 2.4. Strategy group was treated as a nominal five-level exposure. Because this was a retrospective cohort study, no prospective sample-size or post hoc power calculation was performed. Unless otherwise stated, results were reported with two-sided 95% confidence intervals (CIs).

Continuous variables were summarized as the mean and standard deviation or the median and interquartile range, according to distributional shape; categorical variables were summarized as counts and percentages among assessable dogs. Baseline characteristics were described without null-hypothesis significance testing. Imbalance was assessed using standardized mean differences (SMDs), summarized for each variable as the maximum absolute SMD across the ten pairwise group comparisons; values ≥ 0.10 were considered potentially meaningful for descriptive purposes. The breed was described but was not modeled because of sparse categories.

The primary outcome was the joint target-time percentage defined in Section 2.7, and the primary estimand was the covariate-adjusted between-group difference in mean percentage points. Unadjusted and adjusted group means and differences were estimated using linear regression with heteroscedasticity-consistent HC3 standard errors. The adjusted model included age, body weight, sex/neuter status, ASA physical status, cardiac status, renal or respiratory risk, urgency, surgical stratum, procedural complexity, and anesthetist code. Age and weight were modeled continuously and the remaining covariates categorically. All covariates in the principal model were prespecified and retained without univariable screening or stepwise selection; post-strategy variables and primary-window duration were excluded from the principal model. The overall strategy-group effect was assessed using a four-degree-of-freedom robust Wald test, and adjusted means were obtained by marginal standardization over the observed study population.

Five primary contrasts were prespecified: C versus A (integrated rocuronium–sugammadex–controlled ventilation versus conventional spontaneous ventilation), B versus A (planned controlled ventilation without an NMBA versus conventional spontaneous ventilation), C versus B (the rocuronium-containing strategy under planned controlled ventilation, with later planned sugammadex reversal), C versus D (rocuronium versus vecuronium under controlled ventilation and sugammadex reversal), and D versus E (sugammadex versus neostigmine–atropine after vecuronium). For outcomes measured before administration of the reversal agent, contrasts involving the planned reversal component were interpreted as comparisons between initially selected strategies rather than as causal effects of the subsequently administered reversal drug. Accordingly, interpretation of the D-versus-E reversal-agent contrast was restricted to post-reversal quantitative recovery and emergence outcomes. Effects were calculated as the first-named group minus the second-named group. Positive differences favored the first-named group for target-time percentages, whereas negative differences favored it for burdens, recorded anesthetic exposures, adverse-event risks, and recovery times. Family-wise type I error across the five prespecified contrasts was controlled at 0.05 using a single-step max-|Z| adjustment based on their joint covariance matrix; multiplicity-adjusted *p* values and simultaneous 95% CIs were reported irrespective of the omnibus result. All ten pairwise group comparisons were additionally reported as exploratory comparisons using the same single-step max-|Z| procedure applied to the complete ten-contrast joint covariance matrix. The multivariate-normal reference distribution was approximated using 2,000,000 pseudo-random draws with prespecified fixed seeds.

Unless otherwise specified, secondary continuous outcomes used the same baseline adjustment set and marginal-standardization approach. EtCO_2_ and MAP target-time percentages, time-weighted mean end-tidal isoflurane concentration, time-weighted mean fentanyl infusion rate, and extubation TOFR were analyzed using HC3-robust linear regression. EtCO_2_ dysregulation burden, hypotension burden, inhalant-discontinuation-to-extubation time, and reversal-related recovery times were analyzed using gamma generalized linear models with log links. Adjusted marginal means were reported for all outcomes. Effects were expressed as absolute differences for identity-link models; for log-link models, ratios of adjusted means were the principal contrast scale, with absolute differences additionally reported for the physiologic burden outcomes. The paired coverage and controlled-ventilation percentage were interpreted as data-completeness and strategy-implementation measures, respectively. Ventilator-setting changes were analyzed using negative-binomial regression, with primary-window duration included as a prespecified proxy covariate for observation time. This analysis was considered exploratory. Strategy contrasts from this count model were adjusted using the same single-step max-|Z| procedure specified for the five-group continuous outcomes.

Reversal and quantitative neuromuscular-recovery outcomes were analyzed only in Groups C–E, as specified in Section 2.9 and Section 2.10. Their principal comparisons were C versus D and D versus E; C versus E was exploratory. TOFC at reversal and NMBA and reversal doses were summarized descriptively. TOFR at reversal was not analyzed as an ordinary continuous outcome when TOFC was 0–3 and was summarized descriptively by strategy group and TOFC category. In addition to models estimating the total strategy association, supportive recovery-condition models adjusted for baseline TOFR, TOFC at reversal, esophageal temperature and end-tidal isoflurane concentration at reversal, and the interval from the final NMBA dose to reversal. The supportive D-versus-E analysis was restricted to dogs with TOFC 2–4 and additionally adjusted for cumulative vecuronium dose.

Ventilator asynchrony was analyzed among Groups B–E; structurally inapplicable Group A observations were excluded. Unplanned ventilatory intervention was analyzed as any qualifying intervention versus none, with the intervention type summarized descriptively. Rescue anticholinergic use was analyzed as any additional atropine or glycopyrrolate administration versus none, with the individual agents summarized descriptively. Binary management outcomes defined in Section 2.5 and safety outcomes defined in Section 2.11 were reported as events per assessable dogs with exact binomial 95% CIs. Overall comparisons used the Fisher–Freeman–Halton exact test when any expected cell count was <5 and Pearson’s chi-square test otherwise; pairwise comparisons used two-sided Fisher exact tests with Holm adjustment. Risks, risk differences, and risk ratios were reported. Group-specific risk CIs were calculated using the Clopper–Pearson exact method, unadjusted risk-difference CIs using the Newcombe hybrid-score method, and unadjusted risk-ratio CIs using the Katz log method. When a zero-event cell made a risk ratio undefined, it was reported as not estimable, and no continuity correction was applied.

Covariate-adjusted binary analyses were considered exploratory and were undertaken only when at least 40 events occurred overall and every applicable strategy group contained at least one event. Modified Poisson regression with robust standard errors was used to estimate adjusted risk ratios, probabilities, and risk differences. The parsimonious adjustment set comprised age as a continuous term; ASA III–IV versus II; clinically significant cardiac disease or a history of congestive heart failure versus none or subclinical disease; any renal or respiratory risk versus none; urgent or emergency versus elective presentation; thoracolumbar IVDE versus orthopedic surgery; procedural complexity coded ordinally as S1 = 1, S2 = 2, and S3 = 3; and anesthetist code. Bias-reduced Firth logistic regression was reserved for complete or quasi-complete separation, in which case odds ratios and profile penalized-likelihood 95% CIs were reported. Model convergence, influential observations, and the precision of adjusted estimates were examined before interpretation. Out-of-range individual fitted probabilities were treated as model-adequacy warnings rather than as an automatic indication of model failure. When all marginal standardized risks remained within the 0–1 probability range and the model otherwise produced finite, stable estimates, model-based adjusted risks, risk differences, and risk ratios were retained only as exploratory supportive estimates. When boundary violations were accompanied by high leverage or other evidence of instability, adjusted estimates were not interpreted. The observed exact analyses remained principal for these binary outcomes. If a model failed to converge or produced nonfinite estimates, the adjusted effect was reported as not estimable, and interpretation was restricted to the observed event rates, exact comparisons, and unadjusted effect estimates. Outcomes with 10–39 events were limited to exact and unadjusted comparisons, and those with fewer than 10 events were reported descriptively. Hypoxemia observations classified as ‘Not assessable’ were excluded from the principal analysis and examined in boundary-case sensitivity analyses by alternatively classifying all such observations as events or nonevents. Themaximum dopamine dose was summarized among dopamine recipients only.

For secondary outcomes, omnibus *p* values were adjusted using the Benjamini–Hochberg false-discovery-rate procedure within four prespecified domains: physiologic performance (EtCO_2_ target-time percentage, MAP target-time percentage, EtCO_2_ dysregulation burden, and hypotension burden); anesthetic and ventilatory management (time-weighted mean end-tidal isoflurane concentration, time-weighted mean fentanyl infusion rate, ventilator asynchrony, unplanned ventilatory intervention, and ventilator-setting changes); neuromuscular recovery (time to normalized TOFR ≥ 0.90, time to normalized TOFR ≥ 1.00, extubation TOFR, reversal-to-extubation time, and inhalant-discontinuation-to-extubation time); and peri-anesthetic safety (dopamine use, fluid-bolus use, rescue-anticholinergic use, bradyarrhythmia, hypoxemia, early respiratory events, major >3–12 h events, and hard 48 h events). Paired coverage, controlled-ventilation percentage, descriptive drug- and dose-related measures, and epinephrine use were excluded from multiplicity adjustment. For five-group continuous outcomes, single-step max-|Z| adjustment was applied separately to the five prespecified contrasts and to the exploratory family of all ten pairwise comparisons. For continuous outcomes restricted to Groups C–E, the applicable C–D, D–E, and C–E contrasts were adjusted jointly within each outcome. Exact binary comparisons were Holm-adjusted over all applicable pairwise comparisons within each outcome, comprising ten comparisons for five-group outcomes and six comparisons for ventilator asynchrony. Secondary pairwise analyses were interpreted as supportive or exploratory, with an emphasis on effect sizes, CIs, and consistency across related endpoints. For clinical interpretation, adjusted primary-outcome estimates were displayed jointly with the observed exact risks of the prespecified hard 48 h event and, supportively, early respiratory events within 0–3 h. This descriptive benefit–risk display did not define a new composite endpoint, weighting scheme, formal ranking estimand, or additional hypothesis test.

Sensitivity analyses for the primary outcome comprised exclusion of major crossovers, restriction to paired coverage ≥ 90%, fractional-response regression with a logit link, generalized propensity-score overlap weighting using the principal baseline covariates, supportive adjustment for primary-window duration, and dog-level nonparametric bootstrap CIs based on 2000 resamples. Covariate balance after weighting was assessed using absolute SMDs. Strategy interactions with surgical stratum and with ASA II versus III–IV were examined exploratorily in separate models.

An additional post hoc exploratory analysis examined the association between strategy group and the primary outcome after accounting for concomitant anesthetic exposure. Three models added the time-weighted mean end-tidal isoflurane concentration and fentanyl CRI rate, as defined in Section 2.3, individually and jointly to the principal baseline-adjusted model. Both exposures were modeled continuously, and all original covariates, including anesthetist code as a categorical fixed effect, were retained. All models included the same 288 dogs and used HC3 standard errors. Single-step max-|Z| adjustment provided simultaneous 95% CIs and adjusted *p* values across the five prespecified contrasts within each model, using 2,000,000 multivariate-normal draws with a fixed random seed for reproducibility. No additional multiplicity adjustment was applied across the exploratory model specifications. Exposure correlations, variance-inflation factors, and between-group exposure overlap were examined.

Model adequacy was assessed using residual, influence, variance-inflation, dispersion, and linearity diagnostics; verified influential observations were retained in the principal analysis. Structural inapplicability, undefined measurements, and unrecorded or unassessable values were handled as described in Section 2.9, Section 2.10, Section 2.11 and Section 2.12 and were not imputed; endpoint-specific denominators were reported. The primary outcome and its prespecified adjustment variables were complete, and multiple imputation was therefore not required. Statistical significance for the primary family was defined as a multiplicity-adjusted *p* value < 0.05; secondary adjusted *p* or q values were interpreted at the 0.05 level. Effect estimates and 95% CIs were reported irrespective of significance. Analyses were performed using Python 3.12.13 (Python Software Foundation, Wilmington, DE, USA), with NumPy 2.3.5, pandas 2.2.3, SciPy 1.17.0, and scikit-learn 1.8.0. Custom routines were used for robust covariance estimation, marginal standardization, simultaneous inference, exact comparisons, and bootstrap analyses.

## 3. Results

### 3.1. Study Cohort, Baseline Characteristics, and Data Completeness

**Figure 1:**
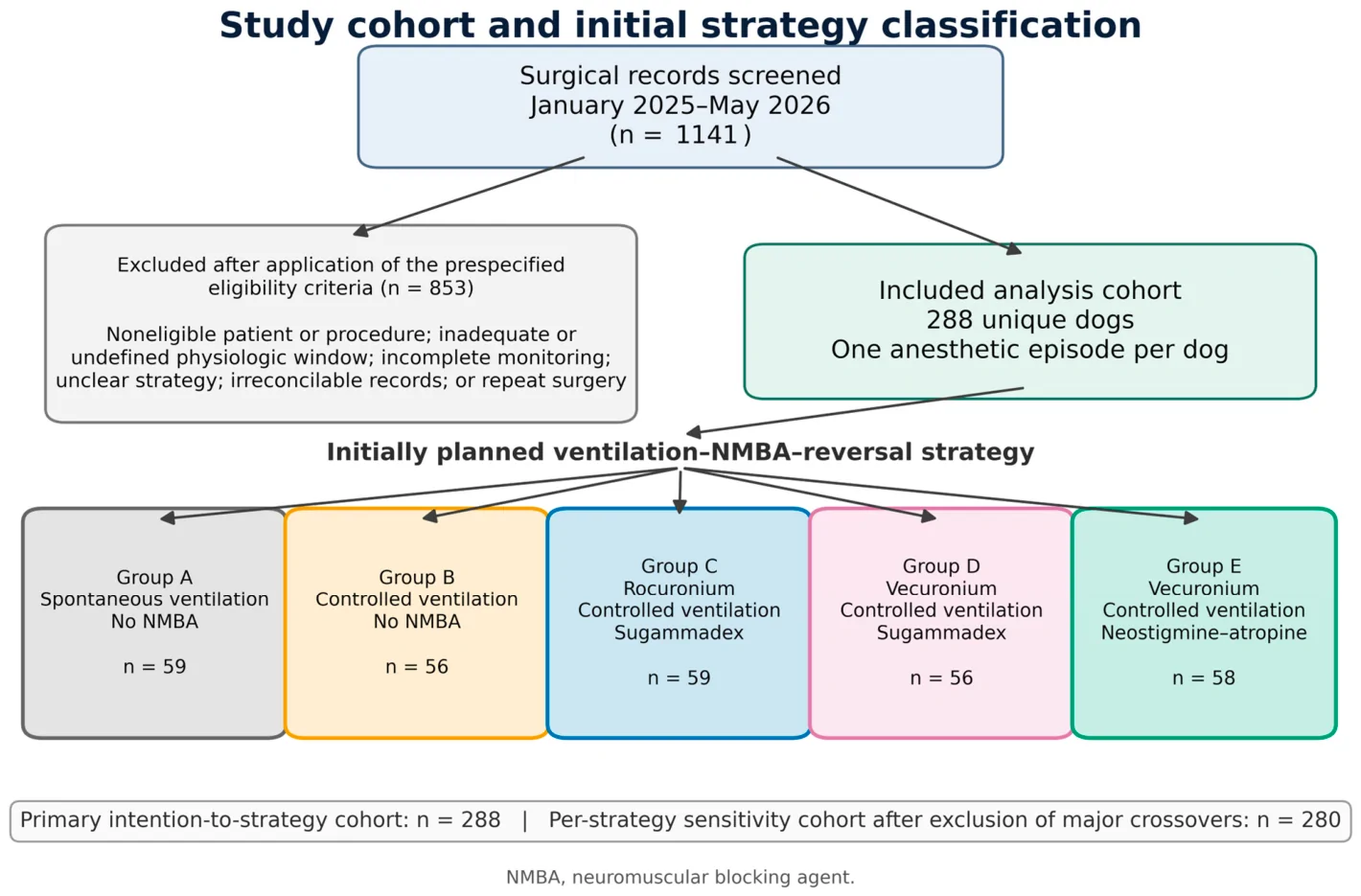
Study flow and classification into the five prespecified anesthetic strategy groups. The primary analysis retained dogs according to the initially planned strategy. Eight major crossovers were excluded only from the per-strategy sensitivity analysis. NMBA, neuromuscular blocking agent.

Between January 2025 and May 2026, 1141 surgical records were screened. Of these, 853 did not meet one or more of the prespecified eligibility criteria, leaving 288 anesthetic episodes from 288 individual dogs for analysis (Figure 1). The cohort comprised 59 dogs in Group A, 56 in Group B, 59 in Group C, 56 in Group D, and 58 in Group E. Orthopedic procedures accounted for 262 cases (91.0%), and thoracolumbar hemilaminectomy for intervertebral disc extrusion accounted for 26 cases (9.0%).

**Figure 2:**
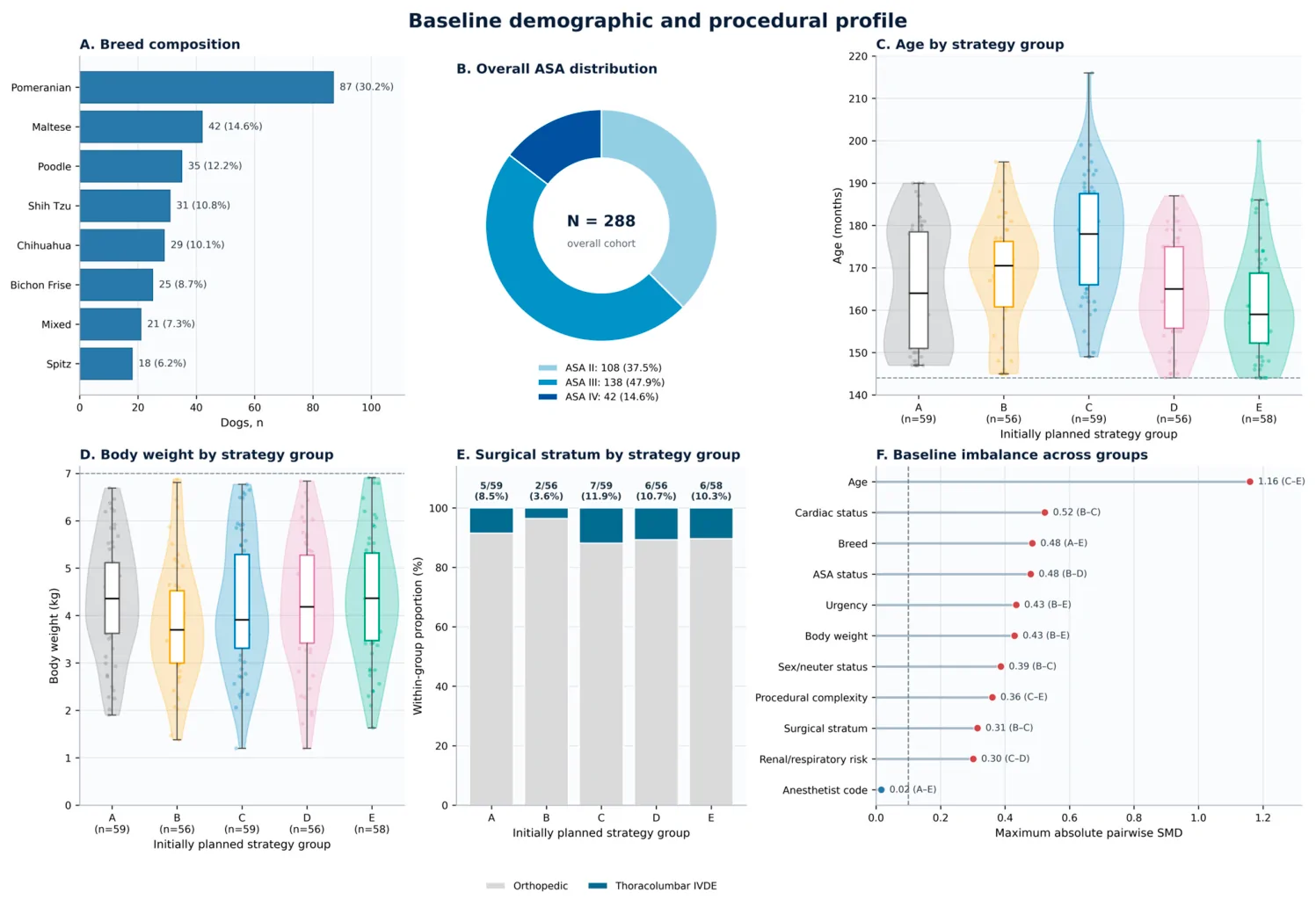
Baseline demographic and procedural profile of the study cohort. (**A**) Overall breed distribution and (**B**) overall American Society of Anesthesiologists (ASA) physical-status distribution among the 288 included dogs. (**C**,**D**) Distributions of age and body weight, respectively, according to the initially planned anesthetic strategy; individual observations are overlaid with density and boxplot summaries showing the median and interquartile range. Horizontal dashed lines denote the eligibility limits of age ≥ 12 years and body weight ≤ 7 kg. (**E**) Distribution of orthopedic procedures and thoracolumbar hemilaminectomy for intervertebral disc extrusion (IVDE) within each strategy group. (**F**) Maximum absolute standardized mean difference (SMD) for each baseline characteristic across the ten pairwise strategy-group comparisons. The vertical dashed line at an absolute SMD of 0.10 is a descriptive reference for potentially meaningful imbalance, not a hypothesis-test significance threshold. All panels show unadjusted descriptive data; strategy groups were clinical exposure groups and were not randomized.

The mean age was 167.2 months (standard deviation [SD], 14.2; range, 144–216), and the mean body weight was 4.21 kg (SD, 1.30; range, 1.20–6.91). ASA physical status was II in 108 dogs (37.5%), III in 138 (47.9%), and IV in 42 (14.6%). Procedures were classified as elective in 166 dogs (57.6%), urgent in 91 (31.6%), and emergency in 31 (10.8%). Baseline characteristics according to the initially planned strategy are presented in Table 1 and Figure 2. The largest baseline imbalances involved age (maximum absolute pairwise standardized mean difference [SMD], 1.16), cardiac status (0.52), breed (0.48), ASA physical status (0.48), body weight (0.43), and urgency (0.43). Surgical-stratum imbalance was smaller (0.31), and anesthetist-code distribution was closely aligned across groups (0.02). Descriptively, Group C had the oldest age distribution and the highest proportion of dogs with clinically significant cardiac disease or a history of congestive heart failure (25/59, 42.4%), whereas urgent or emergency presentations were most frequent in Group B (28/56, 50.0%).

The primary outcome and all prespecified baseline adjustment variables were complete for all 288 dogs. The mean primary physiologic-window duration was 147.4 min (SD, 49.2; range, 60.0–273.0), and mean paired MAP–EtCO_2_ coverage was 94.2% (SD, 3.2; range, 84.8–100.0). All included cases exceeded the prespecified minimum paired-coverage threshold of 70%, and 258 dogs (89.6%) had a paired coverage of at least 90%. Follow-up through 48 h after extubation was available for all dogs. Pre-extubation hypoxemia was assessable in 283 of 288 dogs (98.3%); the remaining five observations were classified as not assessable. Neuromuscular-block and reversal outcomes were structurally applicable to the 173 dogs in Groups C–E, and ventilator asynchrony was structurally applicable to the 229 dogs in Groups B–E; structural nonapplicability was not treated as missing data.

Eight dogs (2.8% of the analysis cohort), all initially classified in Group A, met the prespecified major-crossover criterion because controlled-ventilation exposure exceeded 20% of the post-intubation ventilatory-assessment window. These dogs remained in Group A for the primary intention-to-strategy analysis; their exclusion yielded a per-strategy sensitivity cohort of 280 dogs.

### 3.2. Primary Physiologic Outcome: Joint Target-Time Percentage

**Figure 3:**
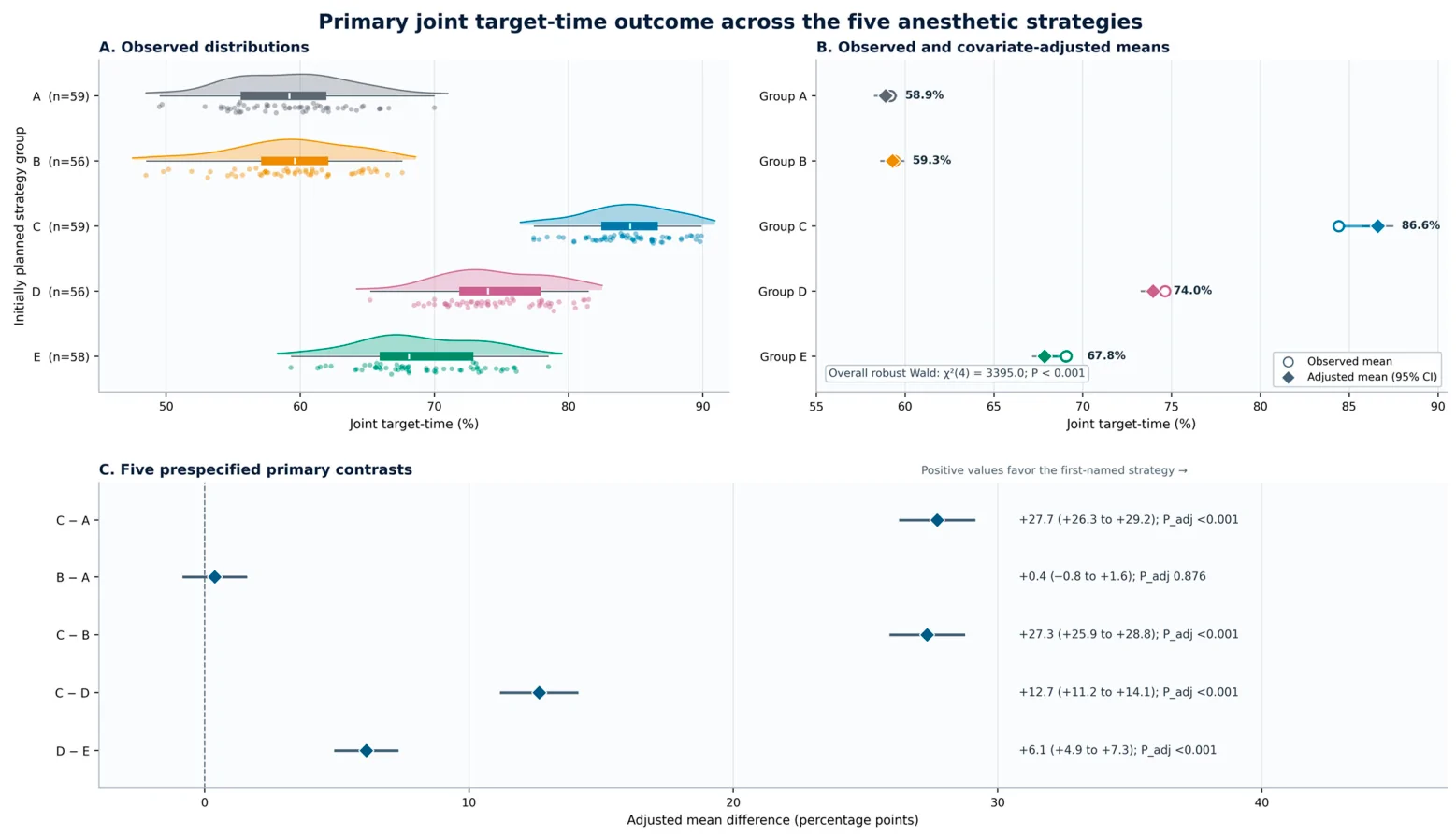
Observed and adjusted primary joint target-time outcome across the five initially planned anesthetic strategies. (**A**) Horizontal raincloud plots show kernel-density distributions, individual dogs, medians, interquartile ranges, and observed ranges. (**B**) Open circles denote observed means; filled diamonds denote covariate-adjusted marginal means, horizontal lines denote pointwise 95% confidence intervals, and connectors show the extent of adjustment. Marginal means were standardized over the observed study population using the prespecified HC3 linear model. (**C**) Diamonds show adjusted differences for the five prespecified contrasts, calculated as the first-named group minus the second-named group; horizontal lines denote simultaneous 95% confidence intervals, and *p* values were adjusted across the five-contrast family using the prespecified single-step procedure. Positive values favor the first-named group. Group sizes were A, *n* = 59; B, *n* = 56; C, *n* = 59; D, *n* = 56; and E, *n* = 58. CI, confidence interval; pp, percentage points.

The observed mean (standard deviation [SD]) joint target-time percentage was 59.2% (4.2) in Group A, 59.4% (4.4) in Group B, 84.4% (3.2) in Group C, 74.6% (3.8) in Group D, and 69.0% (4.4) in Group E (Figure 3A). After adjustment for the prespecified baseline covariates, Group C retained the highest marginal mean (86.6%; 95% confidence interval [CI], 85.7–87.5), followed by Group D (74.0%; 95% CI, 73.3–74.7), Group E (67.8%; 95% CI, 67.1–68.6), Group B (59.3%; 95% CI, 58.6–60.0), and Group A (58.9%; 95% CI, 58.3–59.5) (Figure 3B and Table 2). The adjusted strategy-group association was strong (HC3-robust Wald χ^2^ = 3394.97; df = 4; *p* < 0.001).

For the five prespecified contrasts, the adjusted joint target-time percentage was 27.7 percentage points higher in Group C than in Group A (simultaneous 95% CI, 26.3–29.2; single-step adjusted *p* < 0.001) and 27.3 percentage points higher in Group C than in Group B (95% CI, 25.9–28.8; adjusted *p* < 0.001). Groups B and A did not differ clearly (adjusted difference, 0.4 percentage points; 95% CI, −0.8–1.6; adjusted *p* = 0.876). The adjusted value was 12.7 percentage points higher in Group C than in Group D (95% CI, 11.2–14.1; adjusted *p* < 0.001) and 6.1 percentage points higher in Group D than in Group E (95% CI, 4.9–7.3; adjusted *p* < 0.001) (Figure 3C and Table 2).

**Figure 4:**
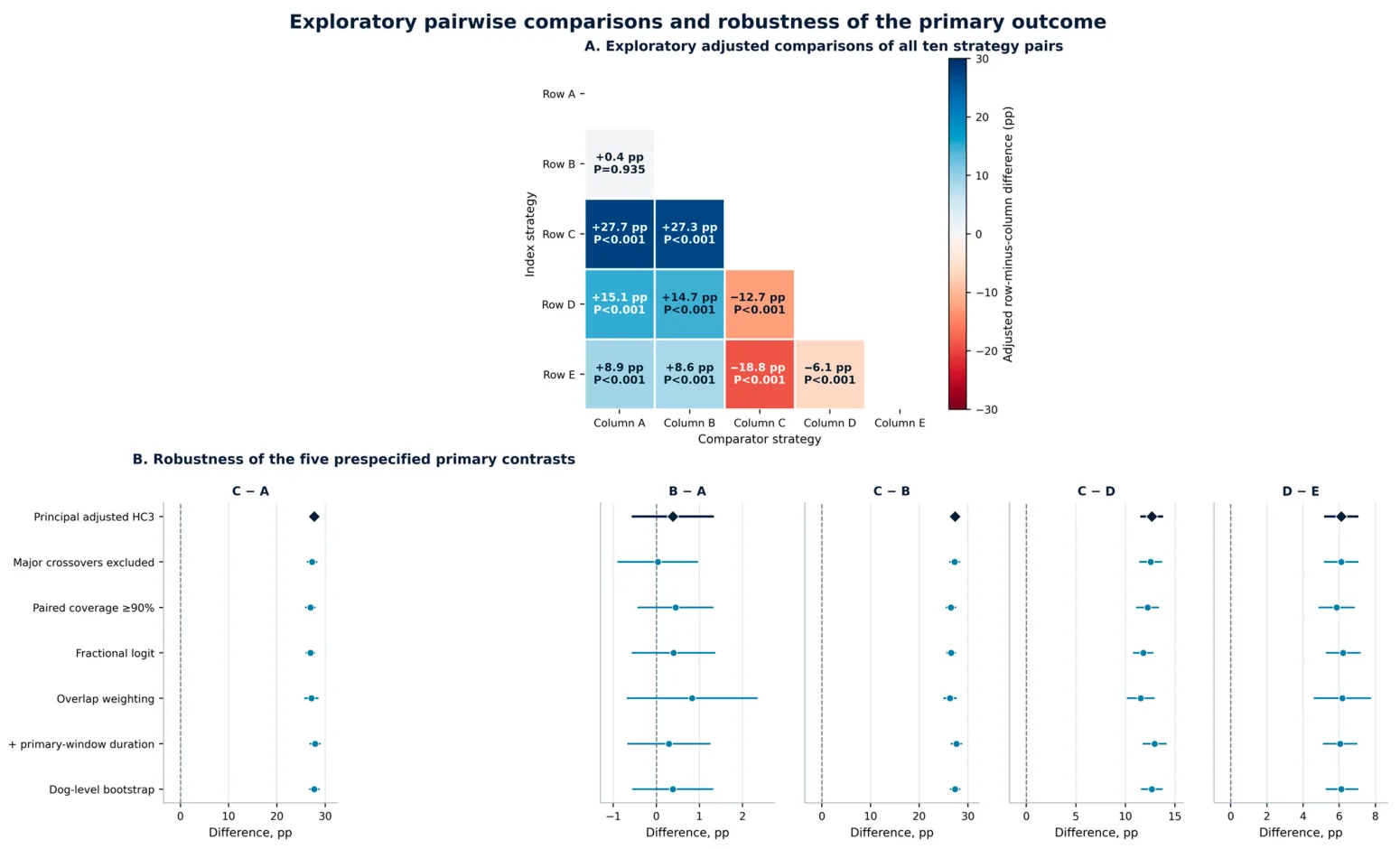
Exploratory pairwise comparisons and sensitivity analyses for the primary joint target-time outcome. (**A**) The lower-triangular matrix displays all ten covariate-adjusted pairwise differences. Each cell is the row strategy minus the column strategy in percentage points; positive values indicate a higher joint target-time percentage in the row strategy. Cell *p* values were adjusted over the ten-comparison family using the joint-covariance single-step max-|Z| procedure. (**B**) Points and horizontal lines show estimates and pointwise 95% confidence intervals for the five prespecified contrasts across sensitivity analyses; the bootstrap row shows percentile 95% confidence intervals from 2000 dog-level resamples. The principal simultaneous inference remains that shown in Figure 3C and Table 2. The overlap-weighted analysis was interpreted as supportive because the maximum post-weighting absolute standardized mean difference was 0.40. pp, percentage points.

The exploratory analysis of all ten adjusted pairwise comparisons showed the same ordered pattern. Simultaneous confidence intervals from the joint-covariance single-step max-|Z| procedure excluded zero for every pair except Groups B and A; this comparison remained small and imprecise after adjustment for the ten-comparison family (B minus A, 0.4 percentage points; single-step adjusted *p* = 0.935) (Figure 4A).

The five prespecified contrast estimates were materially unchanged after exclusion of the eight major crossovers, restriction to dogs with paired coverage of at least 90%, fractional-response modeling, generalized propensity-score overlap weighting, additional adjustment for primary-window duration, and dog-level bootstrap resampling (Figure 4B). Across these analyses, estimates ranged from 26.9 to 27.9 percentage points for C versus A, 0.0 to 0.8 for B versus A, 26.3 to 27.6 for C versus B, 11.5 to 12.9 for C versus D, and 5.9 to 6.2 for D versus E. Overlap weighting produced effects in the same direction, although the maximum post-weighting absolute standardized mean difference remained 0.40. There was no clear evidence of effect modification by surgical stratum (interaction *p* = 0.585) or by ASA II versus III–IV status (interaction *p* = 0.564); these interaction analyses were exploratory, and the thoracolumbar-IVDE subgroup was small.

Additional adjustment for both anesthetic exposures attenuated the between-strategy differences, but Group C retained higher joint target-time percentages than Groups A, B, and D: adjusted differences were 14.6 percentage points (simultaneous 95% CI, 11.9–17.3), 9.0 (5.0–13.1), and 6.7 (5.1–8.3), respectively. The B-versus-A estimate increased from 0.4 to 5.6 percentage points (3.1–8.0), and the D-versus-E estimate was 4.5 percentage points (3.5–5.6). All five contrasts in the jointly adjusted model had single-step adjusted *p* values < 0.001. Models adding either exposure separately also retained positive C-versus-A, C-versus-B, and C-versus-D associations. Across the cohort, the two exposures were strongly correlated (r = 0.944), with variance-inflation factors of 47.0 for EtISO and 41.5 for fentanyl CRI in the jointly adjusted model.

### 3.3. Component Physiologic Target Attainment and Dysregulation Burdens

**Figure 5:**
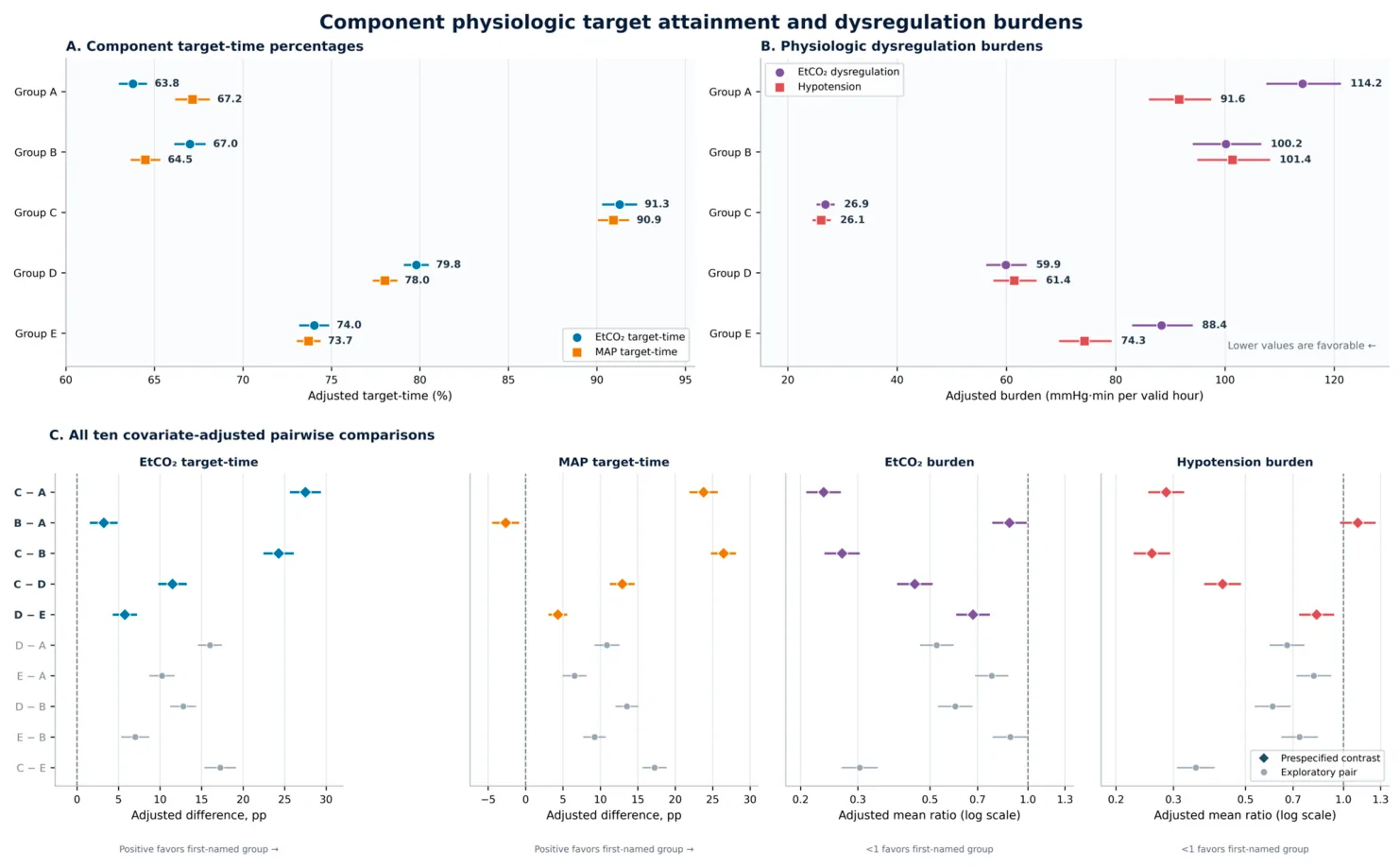
Component physiologic target attainment and dysregulation burdens according to the initially planned anesthetic strategy. (**A**) Covariate-adjusted marginal mean EtCO_2_ target-time and MAP target-time percentages; (**B**) covariate-adjusted marginal mean EtCO_2_ dysregulation and hypotension burdens. Points denote standardized marginal means, and horizontal lines denote pointwise 95% confidence intervals. (**C**) All ten adjusted pairwise comparisons. Target-time effects are first-named minus second-named group in percentage points; positive values favor the first-named group. Burden effects are first-named divided by second-named adjusted means; ratios < 1 favor the first-named group. Adjusted mean-ratio axes in panel C are displayed on logarithmic scales. Diamonds identify the five prespecified clinical contrasts and circles identify the remaining exploratory pairs. Horizontal lines in panel C denote simultaneous 95% confidence intervals from the joint-covariance single-step max-|Z| procedure within each ten-comparison outcome family. Target-time outcomes were estimated with HC3-robust linear regression, and burden outcomes with gamma generalized linear models using log links; both used the prespecified baseline adjustment set and marginal standardization. Omnibus *p* values for the four outcomes were Benjamini–Hochberg adjusted within the physiologic-performance domain and yielded q < 0.001 for each. Case-level data and variable definitions are provided in Supplementary Table S1. CI, confidence interval; EtCO_2_, end-tidal carbon dioxide tension; MAP, mean arterial pressure; pp, percentage points.

All four component physiologic outcomes differed across the five initially planned strategies after false-discovery-rate adjustment within the physiologic-performance domain (FDR-adjusted q < 0.001 for each outcome; Figure 5). Case-level values and variable definitions are provided in Supplementary Table S1.

The adjusted EtCO_2_ target-time percentages were 63.8% in Group A, 67.0% in Group B, 91.3% in Group C, 79.8% in Group D, and 74.0% in Group E (Figure 5A). Under the joint-covariance single-step max-|Z| procedure, Group C exceeded Group A by 27.5 percentage points (simultaneous 95% CI, 25.6–29.4), Group B exceeded Group A by 3.2 percentage points (95% CI, 1.5–4.9), Group C exceeded Group B by 24.3 percentage points (95% CI, 22.4–26.1) and Group D by 11.5 percentage points (95% CI, 9.8–13.2), and Group D exceeded Group E by 5.8 percentage points (95% CI, 4.3–7.2); all single-step adjusted *p* < 0.001.

The adjusted EtCO_2_ dysregulation burden was lowest in Group C (26.9 mmHg·min per valid hour), compared with 114.2 in Group A, 100.2 in Group B, 59.9 in Group D, and 88.4 in Group E (Figure 5B). The absolute difference and adjusted mean ratio were −87.3 mmHg·min/h (simultaneous 95% CI, −97.1 to −77.6) and 0.24 (95% CI, 0.21–0.27) for C versus A; −14.0 (95% CI, −26.9 to −1.1) and 0.88 (95% CI, 0.78–0.99) for B versus A; −73.3 (95% CI, −82.3 to −64.3) and 0.27 (95% CI, 0.24–0.30) for C versus B; −33.0 (95% CI, −38.7 to −27.3) and 0.45 (95% CI, 0.40–0.51) for C versus D; and −28.5 (95% CI, −37.4 to −19.5) and 0.68 (95% CI, 0.60–0.76) for D versus E. The single-step adjusted *p* value was 0.028 for B versus A and <0.001 for the other four prespecified comparisons.

The adjusted MAP target-time percentages were 67.2% in Group A, 64.5% in Group B, 90.9% in Group C, 78.0% in Group D, and 73.7% in Group E (Figure 5A). Group C exceeded Group A by 23.8 percentage points (simultaneous 95% CI, 21.9–25.7), Group B was 2.7 percentage points lower than Group A (95% CI, −4.5 to −0.9), Group C exceeded Group B by 26.4 percentage points (95% CI, 24.8–28.1) and Group D by 12.9 percentage points (95% CI, 11.3–14.6), and Group D exceeded Group E by 4.3 percentage points (95% CI, 3.0–5.6); all single-step adjusted *p* < 0.001.

The adjusted hypotension burden was 91.6 mmHg·min per valid hour in Group A, 101.4 in Group B, 26.1 in Group C, 61.4 in Group D, and 74.3 in Group E (Figure 5B). The absolute difference and adjusted mean ratio were −65.5 mmHg·min/h (simultaneous 95% CI, −73.7 to −57.2) and 0.29 (95% CI, 0.25–0.32) for C versus A; 9.8 (95% CI, −2.3 to 21.9) and 1.11 (95% CI, 0.98–1.26) for B versus A; −75.3 (95% CI, −84.8 to −65.8) and 0.26 (95% CI, 0.23–0.29) for C versus B; −35.3 (95% CI, −41.4 to −29.3) and 0.43 (95% CI, 0.37–0.48) for C versus D; and −12.8 (95% CI, −21.2 to −4.5) and 0.83 (95% CI, 0.73–0.94) for D versus E. There was no clear B-versus-A difference (single-step adjusted *p* = 0.184); the other four prespecified comparisons had adjusted *p* < 0.001.

Across the complete ten-comparison family, simultaneous confidence intervals excluded the null for every pairwise comparison of EtCO_2_ target time, MAP target time, and EtCO_2_ dysregulation burden; the E-versus-B EtCO_2_-burden comparison was the least precise (adjusted mean ratio, 0.882; 95% CI, 0.779–0.999; single-step adjusted *p* = 0.047). For the hypotension burden, only B versus A included the null (Figure 5C). Thus, the B-versus-A component pattern was discordant: Group B had a modestly higher EtCO_2_ target time and lower EtCO_2_ burden, but a lower MAP target time and no clear reduction in hypotension burden.

### 3.4. Recorded Anesthetic Exposure and Ventilatory Management

**Figure 6:**
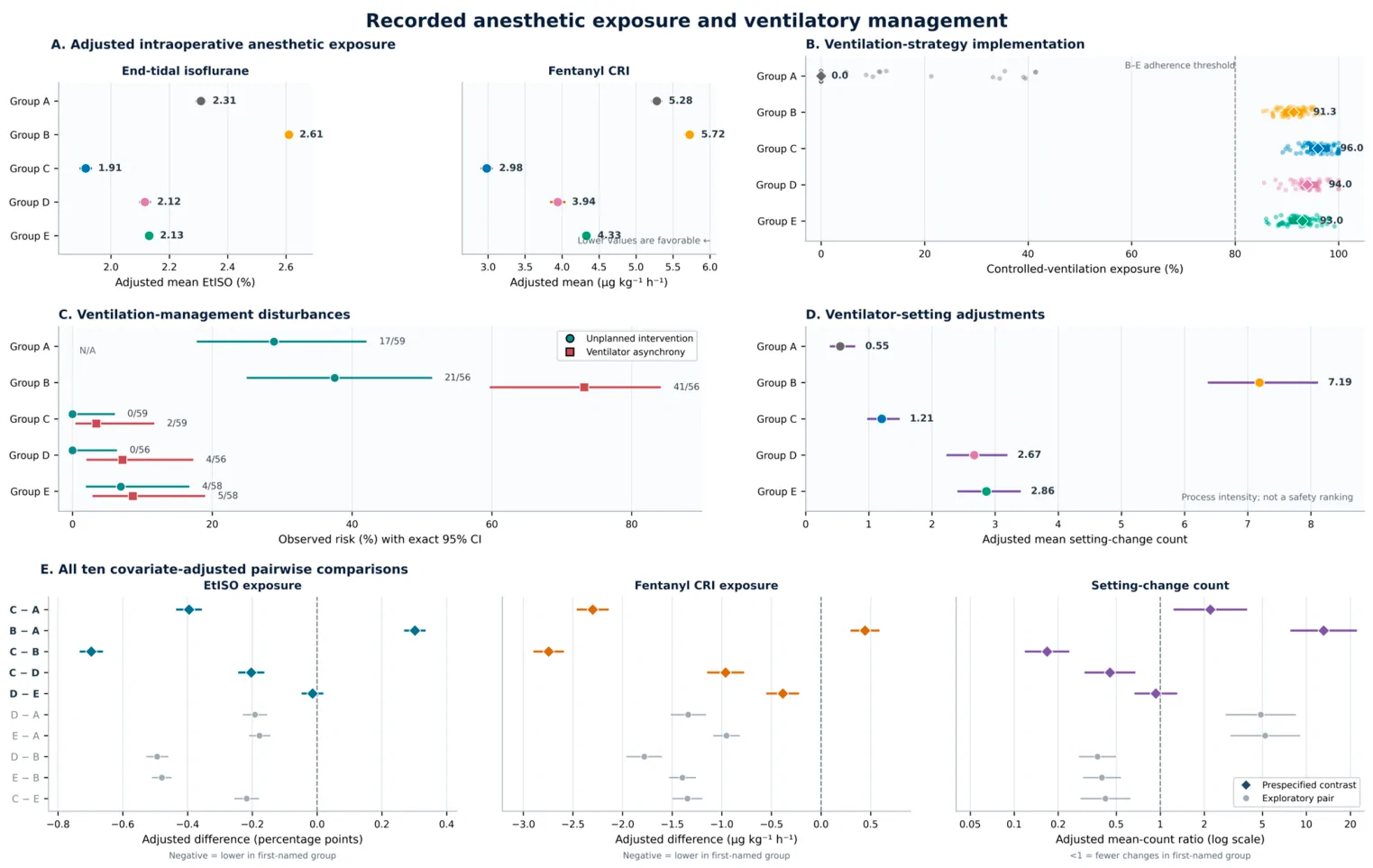
Recorded anesthetic exposure and ventilatory management according to the initially planned strategy. (**A**) Covariate-adjusted marginal mean time-weighted end-tidal isoflurane concentration and fentanyl continuous-infusion rate with pointwise 95% confidence intervals. (**B**) Observed controlled-ventilation exposure; small points represent dogs, thick lines interquartile ranges, and diamonds medians. The dashed line marks the prespecified 80% implementation threshold for Groups B–E. (**C**) Observed risks of ventilator asynchrony and any unplanned ventilatory intervention with exact binomial 95% confidence intervals; Group A asynchrony was structurally not applicable. (**D**) Adjusted marginal mean ventilator-setting-change counts from the exploratory negative-binomial model; estimated dispersion approached the Poisson boundary. (**E**) All ten adjusted pairwise comparisons. Continuous effects are first-named minus second-named group, whereas count effects are adjusted mean ratios. The adjusted mean-ratio axis for setting-change counts in panel E is displayed on a logarithmic scale. Values below the null indicate lower recorded anesthetic exposures or fewer setting changes in the first-named group; setting-change counts do not independently establish safety. Diamonds identify the five prespecified contrasts and circles the remaining exploratory pairs; lines denote simultaneous 95% confidence intervals from the joint-covariance single-step max-|Z| procedure. Models used the prespecified adjustment set and marginal standardization. Case-level observations, definitions, units, and applicability are provided in Supplementary Table S1. CI, confidence interval; CRI, continuous rate infusion; EtISO, end-tidal isoflurane concentration.

All five inferential endpoints in the anesthetic-and-ventilatory-management domain differed among the initially planned strategies after false-discovery-rate adjustment (FDR-adjusted q < 0.001 for each endpoint; Figure 6). Controlled-ventilation exposure was treated as a descriptive strategy-implementation measure and was not included in multiplicity adjustment. Case-level observations and endpoint definitions are provided in Supplementary Table S1.

Adjusted mean end-tidal isoflurane concentrations were 2.31% in Group A, 2.61% in Group B, 1.91% in Group C, 2.12% in Group D, and 2.13% in Group E (robust Wald χ^2^(4) = 3635.78, q < 0.001; Figure 6A). Relative to Group A, Group C was 0.395 percentage points lower (single-step simultaneous 95% CI, −0.435 to −0.355), whereas Group B was 0.302 percentage points higher (95% CI, 0.269–0.335). Group C was 0.697 percentage points lower than Group B (95% CI, −0.733 to −0.661) and 0.203 percentage points lower than Group D (95% CI, −0.244 to −0.163); all four single-step adjusted *p* < 0.001. The D-versus-E difference was −0.014 percentage points (95% CI, −0.048 to 0.019; adjusted *p* = 0.770) and was the only nonsignificant isoflurane comparison in the complete ten-comparison family (Figure 6E).

Adjusted mean fentanyl infusion rates were 5.28 µg kg^−1^ h^−1^ in Group A, 5.72 in Group B, 2.98 in Group C, 3.94 in Group D, and 4.33 in Group E (robust Wald χ^2^(4) = 2818.16, q < 0.001; Figure 6A). The C-versus-A, B-versus-A, C-versus-B, C-versus-D, and D-versus-E adjusted differences were −2.30 (simultaneous 95% CI, −2.46 to −2.14), 0.44 (0.30–0.59), −2.74 (−2.90 to −2.59), −0.96 (−1.15 to −0.77), and −0.39 (−0.55 to −0.22) µg kg^−1^ h^−1^, respectively (all single-step adjusted *p* < 0.001). Every pairwise fentanyl comparison excluded the null (Figure 6E).

Median controlled-ventilation exposure was 0.0% (IQR, 0.0–0.0; range, 0.0–41.5) in Group A, 91.3% (89.4–93.1; 85.4–96.3) in Group B, 96.0% (94.3–98.3; 89.2–100.0) in Group C, 94.0% (92.8–96.1; 85.6–100.0) in Group D, and 93.0% (90.4–94.4; 86.0–99.2) in Group E (Figure 6B). Every dog in Groups B–E had at least 80% controlled-ventilation exposure. Eight Group A dogs exceeded 20%, consistent with the major crossovers described in Section 3.1.

The raw median numbers of ventilator-setting changes were 0 (IQR, 0–1), 7 (6–7.25), 2 (1–2), 2 (1–3), and 2 (1–3) in Groups A–E, respectively. After baseline and primary-window-duration adjustment, the corresponding marginal mean counts were 0.55, 7.19, 1.21, 2.67, and 2.86 (Wald χ^2^(4) = 335.78, q < 0.001; Figure 6D). The adjusted mean-count ratios were 0.168 for C versus B (single-step simultaneous 95% CI, 0.118–0.238) and 0.452 for C versus D (0.302–0.674; both adjusted *p* < 0.001), whereas D versus E did not differ (0.933; 0.665–1.307; adjusted *p* = 0.980). The C-versus-A ratio was 2.20 (1.23–3.93; adjusted *p* = 0.002), but comparisons with Group A reflect structural differences in exposure to planned mechanical ventilation and were not interpreted as safety rankings. The estimated negative-binomial dispersion approached the Poisson boundary; this count analysis was therefore retained as exploratory (Figure 6E).

Ventilator asynchrony occurred in 41/56 dogs (73.2%; exact 95% CI, 59.7–84.2) in Group B, 2/59 (3.4%; 0.4–11.7) in Group C, 4/56 (7.1%; 2.0–17.3) in Group D, and 5/58 (8.6%; 2.9–19.0) in Group E; Group A was structurally not applicable (Pearson χ^2^(3) = 108.22, q < 0.001; Figure 6C). Compared with Group B, Group C had a 69.8-percentage-point lower observed risk (95% CI, 54.6–80.0 points lower) and a risk ratio of 0.046 (95% CI, 0.012–0.182; Holm-adjusted *p* < 0.001). C versus D and D versus E did not differ (adjusted *p* = 0.861 and 1.000, respectively). The prespecified exploratory modified-Poisson model converged, but 13 individual fitted probabilities exceeded 1 (maximum, 2.30) and the maximum leverage was 0.405; because the boundary violations were accompanied by high leverage, adjusted binary estimates were not interpreted.

An unplanned ventilatory intervention occurred in 17/59 dogs (28.8%) in Group A, 21/56 (37.5%) in Group B, 0/59 in Group C, 0/56 in Group D, and 4/58 (6.9%) in Group E (Pearson χ^2^(4) = 55.59, q < 0.001; Figure 6C). Group A events comprised 14 conversions to controlled ventilation and three manual-ventilation episodes; all 21 Group B and all 4 Group E events were manual ventilation. Compared with Group A, Group C had a 28.8-percentage-point lower risk (95% CI, 17.1–41.4 points lower; Holm-adjusted *p* < 0.001), and compared with Group B, it had a 37.5-percentage-point lower risk (95% CI, 24.5–50.6 points lower; adjusted *p* < 0.001). B versus A, C versus D, and D versus E did not differ (adjusted *p* = 0.856, 1.000, and 0.356, respectively). Zero-cell risk ratios were not estimable without continuity correction.

### 3.5. Neuromuscular Blockade, Reversal, and Emergence Recovery

**Figure 7:**
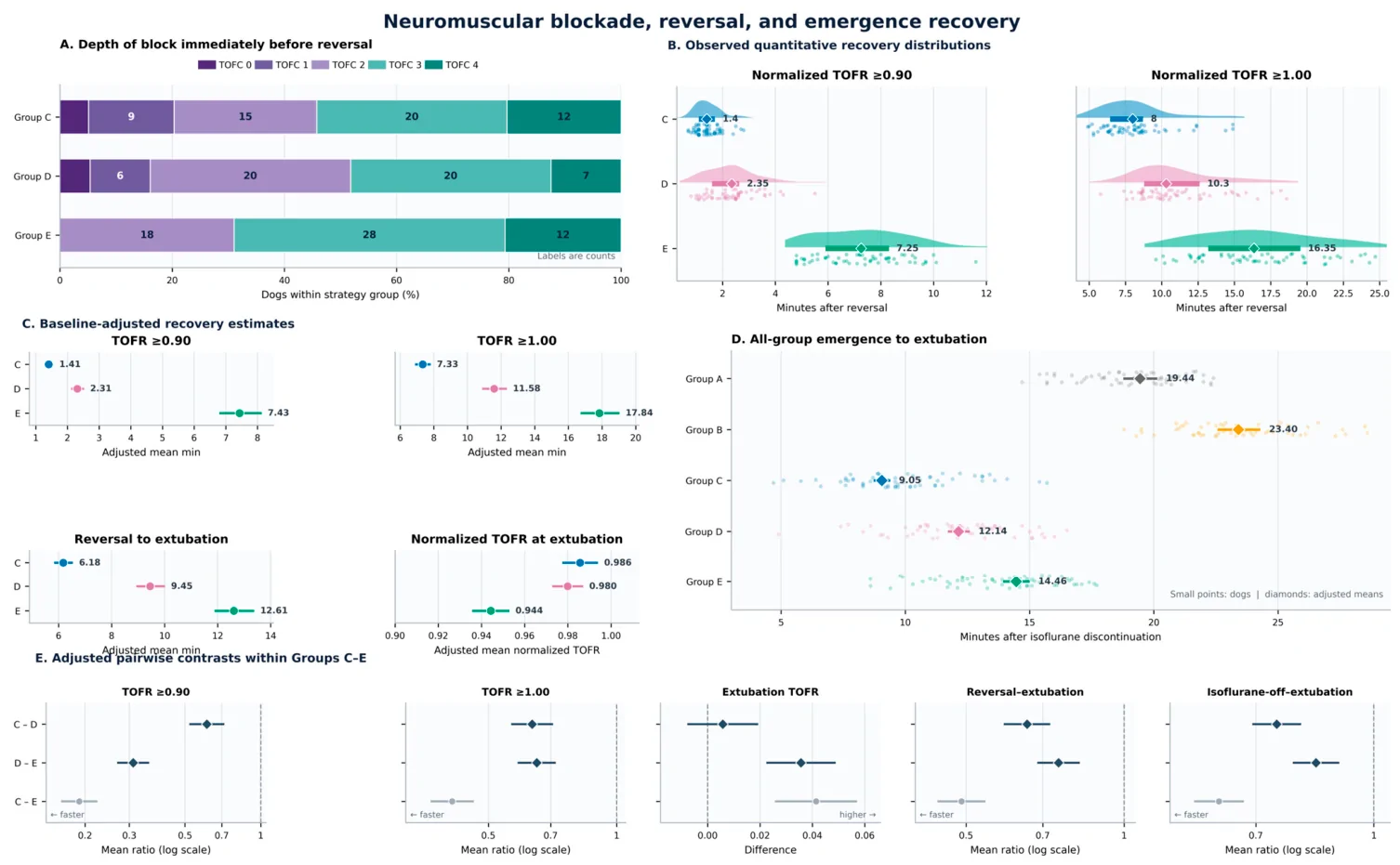
Neuromuscular blockade, reversal, and emergence recovery according to the initially planned strategy. (**A**) Distribution of train-of-four count (TOFC) immediately before reversal; segment labels show numbers of dogs where space permits. (**B**) Observed distributions of elapsed time from reversal to the first of two consecutive normalized train-of-four ratio (TOFR) measurements ≥ 0.90 and ≥1.00. Clouds show kernel density, small points show dogs, thick lines show interquartile ranges, and diamonds show medians. (**C**) Marginal means adjusted for the prespecified baseline covariates with pointwise 95% confidence intervals for the quantitative recovery outcomes in Groups C–E; the dashed line denotes the normalized TOFR 0.90 extubation threshold. (**D**) Observed values and covariate-adjusted marginal mean isoflurane-discontinuation-to-extubation times across all five groups. (**E**) Principal C-versus-D and D-versus-E contrasts (diamonds) and the exploratory C-versus-E contrast (gray circles). Time effects are adjusted mean ratios, for which values < 1 indicate faster recovery in the first-named group; extubation TOFR effects are first-named minus second-named group, for which positive values indicate a higher ratio. Adjusted mean-ratio axes in panel E are displayed on logarithmic scales. Lines in panel E denote simultaneous 95% confidence intervals from the joint-covariance single-step max-|Z| procedure within each outcome. Principal models used the prespecified baseline adjustment set and marginal standardization; recovery-condition supportive models are reported in the text. TOFR at reversal was not plotted as an ordinary continuous measure because it was undefined at TOFC 0 and operationally coded as 0.00 at TOFC 1–3. Case-level data, definitions, units, and group-specific applicability are provided in Supplementary Table S1. CI, confidence interval; NMBA, neuromuscular blocking agent.

Quantitative neuromuscular-monitoring and reversal data were available for all 173 NMBA-treated dogs (Groups C–E), except that TOFR immediately before reversal was undefined as prespecified in six dogs with a TOFC of 0 (three each in Groups C and D). All other quantitative-recovery and emergence endpoints were complete. All five inferential endpoints in the neuromuscular-recovery domain differed by initially planned strategy after false-discovery-rate adjustment (FDR-adjusted q < 0.001 for each endpoint; Figure 7). Case-level observations, definitions, and group-specific applicability are provided in Supplementary Table S1.

The mean (SD) cumulative doses were 0.943 (0.136) mg kg^−1^ for rocuronium in Group C and 0.202 (0.051) and 0.203 (0.046) mg kg^−1^ for vecuronium in Groups D and E, respectively; doses of different NMBAs were summarized descriptively rather than compared inferentially. TOFC values of 0, 1, 2, 3, and 4 immediately before reversal occurred in 3, 9, 15, 20, and 12 dogs in Group C; 3, 6, 20, 20, and 7 in Group D; and 0, 0, 18, 28, and 12 in Group E (Figure 7A). The reversal-dose algorithm was followed in every case: all 21 dogs in Groups C and D with TOFC 0–1 received sugammadex 4 mg kg^−1^, all 94 with TOFC 2–4 received 2 mg kg^−1^, and all Group E dogs received neostigmine only after recovery to TOFC ≥ 2. The mean neostigmine dose was 0.0397 (0.0025) mg kg^−1^ (range, 0.033–0.046).

Mean baseline TOFRs were 0.983, 0.990, and 0.981 in Groups C, D, and E. TOFR at reversal was operationally 0.00 when TOFC was 1–3 and was not treated as an ordinary continuous outcome; among dogs with TOFC 4, median normalized TOFRs were 0.325 (IQR, 0.230–0.393), 0.260 (0.160–0.295), and 0.380 (0.348–0.440), respectively. Median final-NMBA-to-reversal intervals were 25.1, 30.9, and 31.7 min; median esophageal temperatures at reversal were 37.4, 37.1, and 37.0 °C; and median residual end-tidal isoflurane concentrations were 0.70%, 0.82%, and 0.94% in Groups C, D, and E, respectively.

After adjustment for the prespecified baseline covariates, mean times to normalized TOFR ≥ 0.90 were 1.41 min in Group C, 2.31 min in Group D, and 7.43 min in Group E (Wald χ^2^(2) = 622.77, q < 0.001; Figure 7B,C). Recovery was faster in Group C than in Group D (adjusted mean ratio, 0.610; single-step simultaneous 95% CI, 0.520–0.717) and in Group D than in Group E (0.311; 0.268–0.360); both single-step adjusted *p* < 0.001. The exploratory C-versus-E ratio was 0.190 (95% CI, 0.161–0.224; adjusted *p* < 0.001; Figure 7E).

The corresponding adjusted mean times to normalized TOFR ≥ 1.00 were 7.33, 11.58, and 17.84 min in Groups C, D, and E. The adjusted mean ratios were 0.633 for C versus D (simultaneous 95% CI, 0.566–0.709), 0.649 for D versus E (0.586–0.720), and 0.411 for C versus E (0.366–0.462); all adjusted *p* < 0.001 (Figure 7B,C,E). Adjusted mean normalized TOFRs immediately before extubation were 0.986, 0.980, and 0.944. C and D did not differ (difference, 0.0057; simultaneous 95% CI, −0.0078 to 0.0193; adjusted *p* = 0.581), whereas D exceeded E by 0.0356 (0.0224–0.0489; adjusted *p* < 0.001). Nevertheless, every NMBA-treated dog had normalized TOFR ≥ 0.90 before extubation. The TOFR ≥ 1.00 criterion was first documented after extubation in 34/59 dogs (57.6%) in Group C, 36/56 (64.3%) in Group D, and 56/58 (96.6%) in Group E during continued monitoring.

Adjusted mean reversal-to-extubation times were 6.18, 9.45, and 12.61 min in Groups C, D, and E, respectively (Wald χ^2^(2) = 250.98, q < 0.001). The C-versus-D, D-versus-E, and C-versus-E adjusted mean ratios were 0.653 (simultaneous 95% CI, 0.590–0.723), 0.750 (0.683–0.823), and 0.490 (0.441–0.544), respectively; all adjusted *p* < 0.001 (Figure 7C,E).

Across all five strategies, adjusted mean isoflurane-discontinuation-to-extubation times were 19.44 min in Group A, 23.40 in Group B, 9.05 in Group C, 12.14 in Group D, and 14.46 in Group E (Wald χ^2^(4) = 1622.81, q < 0.001; Figure 7D). The adjusted mean ratios for C versus A, B versus A, C versus B, C versus D, and D versus E were 0.466 (single-step simultaneous 95% CI, 0.433–0.500), 1.204 (1.121–1.293), 0.387 (0.359–0.416), 0.746 (0.692–0.803), and 0.840 (0.783–0.901), respectively; all adjusted *p* < 0.001. Every comparison in the complete ten-pair family excluded the null.

The recovery pattern remained after additional adjustment for baseline TOFR, TOFC at reversal, esophageal temperature and end-tidal isoflurane concentration at reversal, and the final-NMBA-to-reversal interval. In the common C–E model, C-versus-D adjusted mean ratios were 0.734 for TOFR ≥ 0.90 recovery (simultaneous 95% CI, 0.610–0.882), 0.710 for TOFR ≥ 1.00 recovery (0.611–0.825), 0.812 for reversal-to-extubation time (0.713–0.925), and 0.874 for isoflurane-discontinuation-to-extubation time (0.794–0.963); the adjusted extubation-TOFR difference remained compatible with no C–D difference (0.0145; −0.0043 to 0.0333). In the prespecified D-versus-E supportive analysis restricted to TOFC 2–4 and additionally adjusted for cumulative vecuronium dose, the corresponding time ratios were 0.315 (95% CI, 0.273–0.363), 0.704 (0.623–0.794), 0.860 (0.779–0.950), and 0.915 (0.844–0.992), and the extubation-TOFR difference was 0.0444 (0.0264–0.0624).

### 3.6. Hemodynamic Support and Pre-Extubation Safety

**Figure 8:**
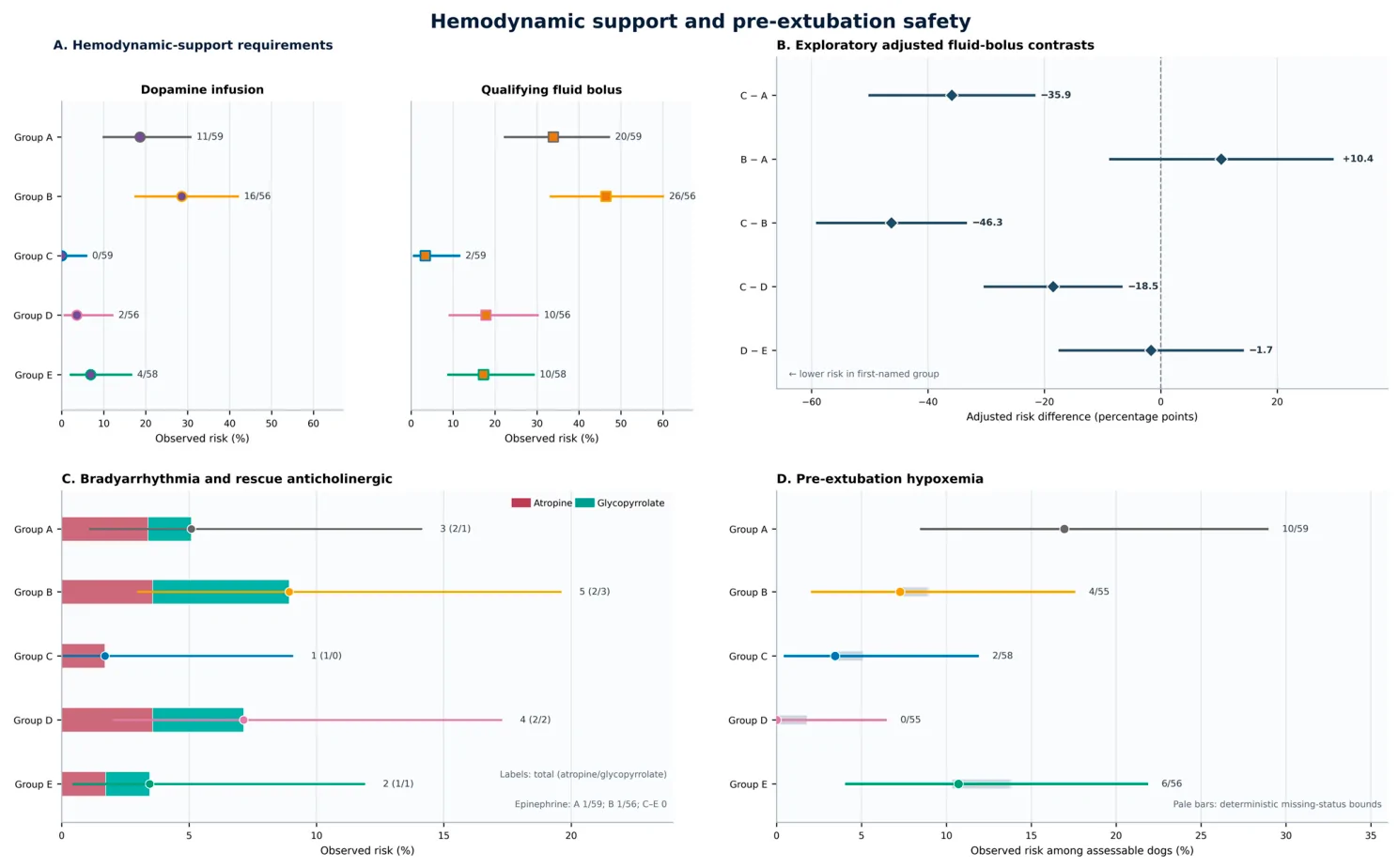
Hemodynamic support and pre-extubation safety according to the initially planned anesthetic strategy. (**A**) Observed risks of dopamine infusion and at least one qualifying crystalloid fluid bolus. Points show group-specific risks, lines show exact binomial 95% confidence intervals (CIs), and labels show events/assessable dogs. (**B**) Exploratory covariate-adjusted risk differences for fluid-bolus use in the five prespecified contrasts. Diamonds show marginal standardized estimates from modified Poisson regression, and lines show pointwise robust 95% CIs; negative values indicate lower risk in the first-named group. Observed exact comparisons remained the principal binary analysis. (**C**) ECG-confirmed bradyarrhythmia and rescue anticholinergic treatment, which occurred in the same 15 dogs. Stacked bars show atropine and glycopyrrolate recipients, points and lines show the total event risk and exact 95% CI, and labels show total events (atropine/glycopyrrolate). Planned atropine coadministered with neostigmine in Group E was excluded from rescue treatment. Epinephrine administration is noted descriptively because only two events occurred. (**D**) Observed pre-extubation hypoxemia risk among dogs with assessable oxygenation data. Points and lines show observed risks and exact binomial 95% CIs; pale bars span deterministic boundary estimates obtained by classifying all five unassessable observations first as nonevents and then as events. Case-level data, definitions, and endpoint-specific denominators are provided in Supplementary Table S1.

Pre-extubation hemodynamic-support and safety outcomes are summarized in Figure 8 and Supplementary Table S1. Dopamine, fluid-bolus, rescue-anticholinergic, epinephrine, and bradyarrhythmia status were complete for all 288 dogs. Hypoxemia was assessable in 283 dogs (98.3%); five observations were classified as not assessable and excluded from its principal analysis.

Dopamine was administered to 11/59 dogs (18.6%) in Group A, 16/56 (28.6%) in Group B, 0/59 in Group C, 2/56 (3.6%) in Group D, and 4/58 (6.9%) in Group E (overall FDR-adjusted q < 0.001; Figure 8A). Use was lower in Group C than in Group A (risk difference, −18.6 percentage points; 95% CI, −30.4 to −8.7; Holm-adjusted *p* = 0.005) and Group B (−28.6 percentage points; −41.5 to −16.7; adjusted *p* < 0.001). Risk ratios for these two contrasts were not estimable because Group C had no events. C did not differ from D or E, and B did not differ from A after adjustment. In the complete ten-pair family, Group B also had greater use than Groups D and E (adjusted *p* = 0.004 and 0.020). Among the 33 recipients, the median maximum infusion rate was 12.3 µg kg^−1^ min^−1^ (IQR, 11.1–13.1; range, 8.7–14.5). Group-specific medians were 12.5, 12.8, 11.3, and 10.2 µg kg^−1^ min^−1^ in Groups A, B, D, and E, respectively; Group C had no recipients.

Qualifying fluid boluses were administered to 20/59 dogs (33.9%) in Group A, 26/56 (46.4%) in Group B, 2/59 (3.4%) in Group C, 10/56 (17.9%) in Group D, and 10/58 (17.2%) in Group E (overall q < 0.001; Figure 8A). Group C had lower observed risks than Groups A and B (risk differences, −30.5 and −43.0 percentage points; 95% CIs, −43.5 to −17.0 and −56.1 to −28.2; Holm-adjusted *p* < 0.001 for both). The corresponding unadjusted risk ratios were 0.10 (95% CI, 0.024–0.409) and 0.073 (0.018–0.293). C-versus-D and D-versus-E differences did not remain significant (adjusted *p* = 0.085 and 1.000). Across all ten comparisons, Group B also exceeded Groups D and E (adjusted *p* = 0.015 and 0.009). The exploratory modified Poisson model converged and yielded standardized fluid-bolus risks of 38.1%, 48.5%, 2.2%, 20.8%, and 22.4% in Groups A–E. Its adjusted risk differences were −35.9 percentage points for C versus A (pointwise robust 95% CI, −50.2 to −21.5), +10.4 for B versus A (−8.9 to 29.7), −46.3 for C versus B (−59.2 to −33.3), −18.5 for C versus D (−30.5 to −6.6), and −1.7 for D versus E (−17.6 to 14.2; Figure 8B); these model-based estimates were treated as supportive. Although all marginal standardized risks were within the probability bounds, four individual fitted values exceeded 1 (maximum, 1.23); these model-based estimates were therefore retained only as exploratory supportive results.

Fifteen ECG-confirmed bradyarrhythmias occurred: 3/59 dogs (5.1%) in Group A, 5/56 (8.9%) in Group B, 1/59 (1.7%) in Group C, 4/56 (7.1%) in Group D, and 2/58 (3.4%) in Group E. Every affected dog received a single intravenous dose of a rescue anticholinergic: atropine 0.02 mg kg^−1^ in eight dogs and glycopyrrolate 0.01 mg kg^−1^ in seven dogs. No repeat rescue anticholinergic doses were administered. Thus, bradyarrhythmia and rescue anticholinergic treatment represented the same cases and were not counted as independent events. Neither bradyarrhythmia nor rescue treatment differed among strategies (both overall q = 0.391; all ten Holm-adjusted pairwise *p* = 1.000; Figure 8C). Epinephrine was administered to two dogs, one each in Groups A and B, and was summarized descriptively.

Pre-extubation hypoxemia occurred in 10/59 assessable dogs (16.9%) in Group A, 4/55 (7.3%) in Group B, 2/58 (3.4%) in Group C, 0/55 in Group D, and 6/56 (10.7%) in Group E (overall q = 0.007; Figure 8D). None of the five prespecified pairwise contrasts was significant after Holm correction (all adjusted *p* ≥ 0.244); in the exploratory complete ten-pair family, only A versus D was significant (adjusted *p* = 0.014). The upper exact 95% confidence limit for the observed zero-event rate in Group D was 6.5%. Overall heterogeneity persisted when all five unassessable observations were classified as nonevents (q = 0.007) or, at the opposite boundary, as events (q = 0.046), whereas no prespecified pairwise contrast was significant in either boundary analysis.

### 3.7. Post-Extubation Respiratory, Major, and Hard Events

Follow-up to 48 h after extubation was completed for all 288 dogs. Early respiratory events within 0–3 h, major cardiopulmonary events occurring >3–12 h, and hard events within 48 h occurred in 75/288 (26.0%), 24/288 (8.3%), and 28/288 dogs (9.7%), respectively. Overall differences among strategies remained after false-discovery-rate adjustment across the prespecified peri-anesthetic safety domain for all three outcomes (q < 0.001, q = 0.010, and q = 0.004, respectively). Group-specific observations and the prespecified composite definitions are provided in Supplementary Table S1.

**Figure 9:**
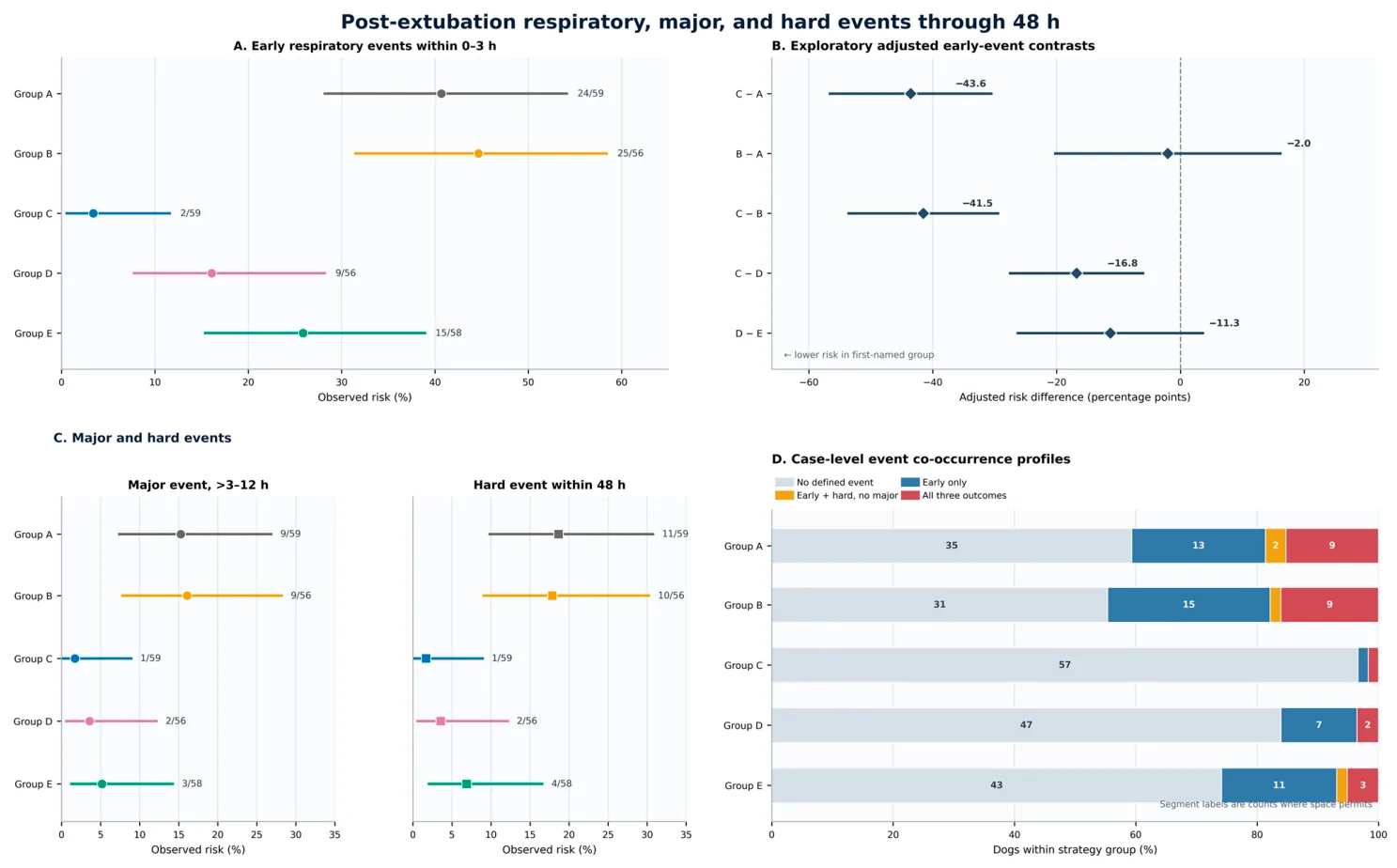
Post-extubation respiratory, major, and hard events according to the initially planned anesthetic strategy. (**A**) Observed risks of an early respiratory event within 0–3 h after extubation. Points denote group-specific risks, horizontal lines denote exact binomial 95% confidence intervals (CIs), and labels give events/number assessed. (**B**) Exploratory covariate-adjusted risk differences for early respiratory events in the five prespecified contrasts, estimated using modified Poisson regression with robust standard errors and marginal standardization. Negative values indicate a lower adjusted probability in the first-named group; horizontal lines denote pointwise robust 95% CIs. Observed exact comparisons remained the principal binary analysis. (**C**) Observed risks of a major event occurring >3–12 h and a hard event occurring within 48 h, with exact binomial 95% CIs and event/denominator labels. (**D**) Mutually exclusive case-level combinations of no postoperative composite, an early event only, early and hard events without a >3–12 h major event, and all three composites. These categories describe observed co-occurrence only and should not be interpreted as causal transitions or validated severity levels. The event windows were prespecified as non-mutually exclusive, and overlapping outcomes were not summed. All panels show unadjusted data except panel B. Omnibus false-discovery-rate-adjusted q values and Holm-adjusted pairwise *p* values are reported in the text. Case-level observations and endpoint definitions are provided in Supplementary Table S1.

Early respiratory events occurred in 24/59 dogs (40.7%) in Group A, 25/56 (44.6%) in Group B, 2/59 (3.4%) in Group C, 9/56 (16.1%) in Group D, and 15/58 (25.9%) in Group E (Figure 9A). In the principal comparisons, Group C had lower observed risks than Groups A and B, with risk differences of −37.3 percentage points (95% CI, −50.3 to −23.1; risk ratio [RR], 0.083; 95% CI, 0.021–0.337; Holm-adjusted *p* < 0.001) and −41.3 percentage points (95% CI, −54.4 to −26.5; RR, 0.076; 95% CI, 0.019–0.306; adjusted *p* < 0.001), respectively. The B-versus-A, C-versus-D, and D-versus-E comparisons did not remain significant (adjusted *p* = 0.709, 0.134, and 0.505, respectively). In the exploratory complete ten-pair family, lower observed risks were also identified for Group D versus Group A (adjusted *p* = 0.024), Group D versus Group B (adjusted *p* = 0.013), and Group C versus Group E (adjusted *p* = 0.004); no other comparison remained significant.

Early respiratory events met the prespecified eligibility criterion for exploratory covariate adjustment. The modified Poisson model converged and yielded marginal standardized risks of 46.0%, 44.0%, 2.4%, 19.2%, and 30.5% in Groups A–E, respectively. Adjusted risk differences were −43.6 percentage points for C versus A (pointwise robust 95% CI, −56.8 to −30.4), −2.0 for B versus A (−20.4 to 16.3), −41.5 for C versus B (−53.8 to −29.3), −16.8 for C versus D (−27.7 to −5.9), and −11.3 for D versus E (−26.5 to 3.8; Figure 9B). Corresponding adjusted RRs were 0.052 (95% CI, 0.013–0.210), 0.956 (0.635–1.438), 0.055 (0.014–0.221), 0.126 (0.029–0.551), and 0.629 (0.331–1.194), respectively. Although all marginal standardized risks were within the probability bounds, 10 individual fitted values exceeded 1 (maximum, 1.90); these model-based estimates were therefore retained only as exploratory supportive results.

Major events occurring >3–12 h after extubation were recorded in 9/59 dogs (15.3%) in Group A, 9/56 (16.1%) in Group B, 1/59 (1.7%) in Group C, 2/56 (3.6%) in Group D, and 3/58 (5.2%) in Group E (Figure 9C). None of the five principal contrasts remained significant after Holm adjustment: adjusted *p* values were 0.149 for C versus A, 1.000 for B versus A, 0.073 for C versus B, 1.000 for C versus D, and 1.000 for D versus E. No additional comparison in the complete ten-pair family was significant.

Hard events within 48 h occurred in 11/59 dogs (18.6%) in Group A, 10/56 (17.9%) in Group B, 1/59 (1.7%) in Group C, 2/56 (3.6%) in Group D, and 4/58 (6.9%) in Group E (Figure 9C). The observed risk was lower in Group C than in Group A (risk difference, −16.9 percentage points; 95% CI, −28.8 to −6.2; RR, 0.091; 95% CI, 0.012–0.682; Holm-adjusted *p* = 0.037) and Group B (−16.2 percentage points; 95% CI, −28.2 to −5.4; RR, 0.095; 95% CI, 0.013–0.718; adjusted *p* = 0.036). The B-versus-A, C-versus-D, and D-versus-E comparisons were not significant (all adjusted *p* = 1.000), and no additional comparison remained significant in the complete pairwise family.

As prespecified, the postoperative event windows were not mutually exclusive. All 28 dogs meeting the hard-event definition also had an early respiratory event, and all 24 dogs with a >3–12 h major event were included among these 28 dogs; four additional dogs had an early and a hard event without a >3–12 h major event. Thus, 75 unique dogs experienced at least one postoperative composite: 47 had an early respiratory event only, 4 had early and hard events without a >3–12 h major event, and 24 met all three composite definitions (Figure 9D). The three outcome counts were therefore not summed.

### 3.8. Integrated Descriptive Comparison of Primary Physiologic Performance and Peri-Anesthetic Risk

**Figure 10:**
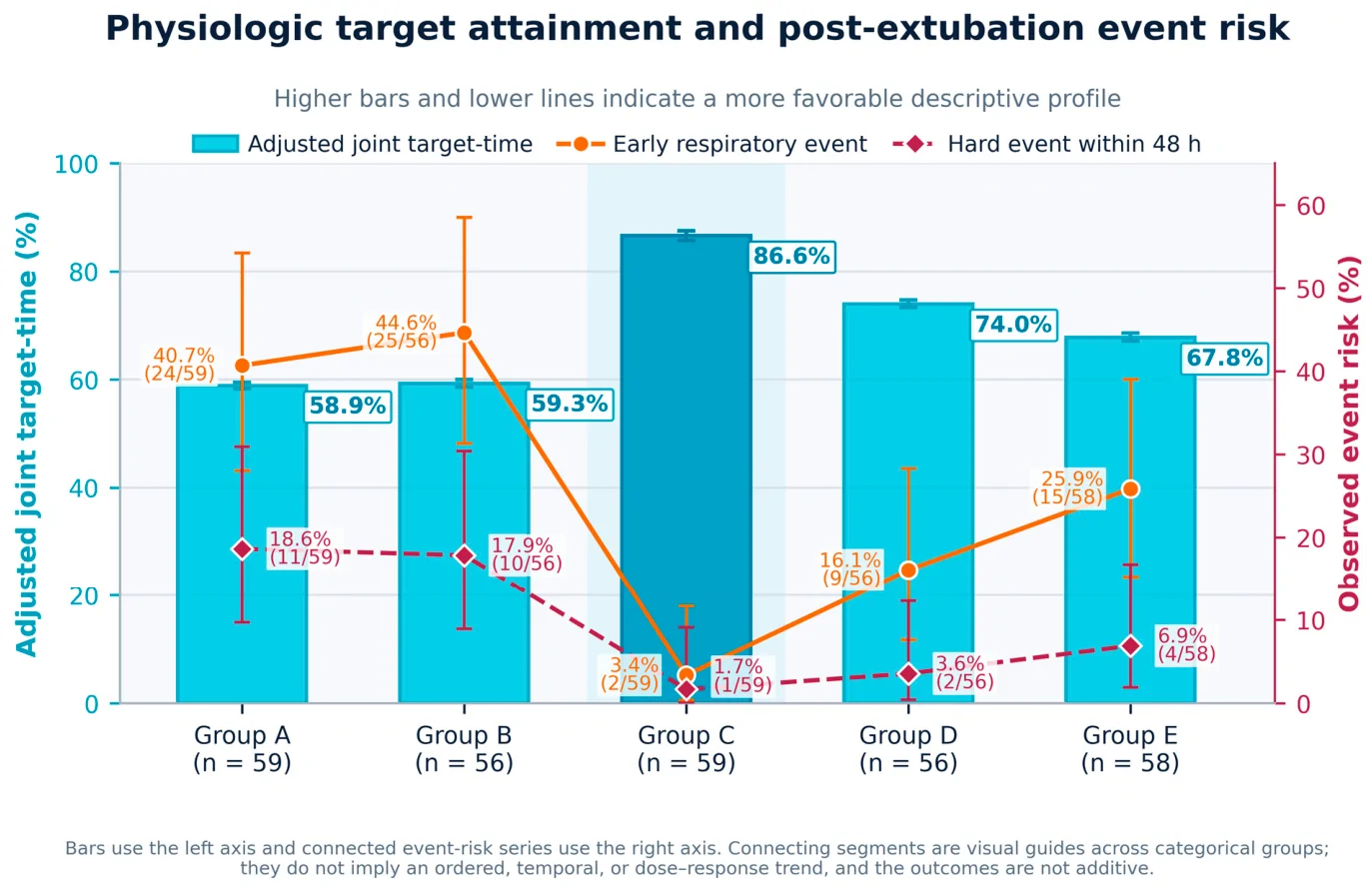
Integrated descriptive comparison of primary physiologic performance and post-extubation event risks according to the initially planned anesthetic strategy. Bars, read against the left *y*-axis, show covariate-adjusted marginal mean joint target-time percentages; vertical error bars denote pointwise 95% confidence intervals (CIs). Connected orange circles, read against the right *y*-axis, show observed risks of an early respiratory event within 0–3 h, and connected red dashed diamonds show observed risks of a hard event within 48 h; vertical error bars denote exact binomial 95% CIs. Event labels give the percentage and number of events/total cases. Higher bars and lower event-risk lines indicate a more favorable descriptive profile. Connecting segments are visual guides across categorical groups and do not imply an ordered, temporal, or dose–response relationship. The estimates were derived using the inferential procedures specified for their respective outcomes and do not constitute a formal joint model. No weighted composite endpoint, scalar benefit-to-risk ratio, or additional ranking test was calculated. Case-level observations and endpoint definitions are provided in Supplementary Table S1.

For integrated clinical interpretation, covariate-adjusted marginal means for the primary outcome were considered jointly with the observed risks of hard 48 h events; early respiratory events within 0–3 h were included as a supportive measure of more frequent postoperative morbidity (Figure 10). This synthesis was descriptive and did not create a weighted composite outcome or an additional hypothesis test. The adjusted joint target-time percentages (95% CIs) were 58.9% (58.3–59.5%) in Group A, 59.3% (58.6–60.0%) in Group B, 86.6% (85.7–87.5%) in Group C, 74.0% (73.3–74.7%) in Group D, and 67.8% (67.1–68.6%) in Group E. In the same group order, the observed risks of a hard event within 48 h were 18.6%, 17.9%, 1.7%, 3.6%, and 6.9%, and those of an early respiratory event were 40.7%, 44.6%, 3.4%, 16.1%, and 25.9%, respectively. Group C therefore combined the highest adjusted primary-outcome estimate with the lowest observed risks for both postoperative event measures.

The principal contrasts supported the same interpretation. Compared with Group A, Group C had a 27.7-percentage-point higher adjusted joint target-time percentage (simultaneous 95% CI, 26.3–29.2; single-step adjusted *p* < 0.001), together with a 16.9-percentage-point lower hard-event risk (95% CI, 6.2–28.8 percentage points lower; Holm-adjusted *p* = 0.037) and a 37.3-percentage-point lower early-event risk (95% CI, 23.1–50.3 percentage points lower; adjusted *p* < 0.001). Compared with Group B, the corresponding differences favoring Group C were 27.3 percentage points for the primary outcome (simultaneous 95% CI, 25.9–28.8; adjusted *p* < 0.001), 16.2 percentage points for hard-event risk (95% CI, 5.4–28.2 percentage points lower; adjusted *p* = 0.036), and 41.3 percentage points for early-event risk (95% CI, 26.5–54.4 percentage points lower; adjusted *p* < 0.001). By contrast, Group B did not differ from Group A in the principal baseline-adjusted analysis of the primary outcome (adjusted difference, 0.4 percentage points; simultaneous 95% CI, −0.8 to 1.6; adjusted *p* = 0.876) and showed no statistically supported reduction in either postoperative event risk.

Group C also exceeded Group D by 12.7 percentage points for the primary outcome (simultaneous 95% CI, 11.2–14.1; adjusted *p* < 0.001); the observed hard- and early-event risks were numerically lower in Group C, although neither principal observed exact contrast remained significant after Holm adjustment. In the exploratory covariate-adjusted analysis, the early-event estimate favored Group C (risk difference, −16.8 percentage points; pointwise 95% CI, −27.7 to −5.9). Group D exceeded Group E by 6.1 percentage points for the primary outcome (simultaneous 95% CI, 4.9–7.3; adjusted *p* < 0.001); the observed postoperative event risks were likewise numerically lower in Group D, although neither principal observed exact D–E safety contrast was statistically significant. Across the outcomes displayed, Group C therefore showed the most favorable integrated descriptive profile: its superior adjusted performance on the prespecified primary physiologic endpoint was accompanied by, rather than traded against, the lowest observed risks for both postoperative event measures. This pattern was also accompanied by low observed use of hemodynamic support, without a statistically supported increase in bradyarrhythmia or hypoxemia. Planned controlled ventilation without neuromuscular blockade (Group B) was not associated with a measurable primary-outcome advantage over conventional spontaneous ventilation (Group A) in the principal baseline-adjusted analysis; observed postoperative event rates were similarly high. These findings support the conclusion that the Group C strategy was associated with superior attainment of the prespecified primary physiologic target in this cohort; however, the integrated interpretation remained descriptive and associative because strategy assignment was not randomized, no formal joint benefit–risk or ranking estimand was specified, and the hard-event estimates were based on observed, non-covariate-adjusted risks.

## 4. Discussion

### 4.1. Principal Findings and Novelty

The principal baseline-adjusted analysis showed that rocuronium-facilitated controlled ventilation was associated with superior concurrent MAP–EtCO_2_ target attainment, whereas controlled ventilation without an NMBA showed no detectable advantage over spontaneous ventilation on this endpoint. This distinction suggests that the physiologic performance of controlled ventilation may depend on how the strategy is implemented, including patient–ventilator interaction and accompanying anesthetic management. Concordant MAP and EtCO_2_ component outcomes support an association with more consistent concurrent physiologic control, without establishing a causal benefit of rocuronium itself.

Although covariate adjustment and the prespecified sensitivity analyses supported the principal associations, the retrospective, nonrandomized design and between-group differences in age, cardiac status, ASA physical status, body weight, and urgency remain important to their interpretation. Patient condition and peri-anesthetic management are central to risk assessment in geriatric dogs [1,2,3,4,6,7]. Consistency across analyses therefore supports the robustness of the observed associations to measured differences, while confounding by indication and unmeasured clinical judgment remain possible.

Previous canine studies have characterized the clinical use, infusion, monitoring, and reversal of neuromuscular blockade predominantly in experimental or relatively small clinical populations [15,16,17,18,19,20,21,23]. The largest earlier direct clinical pharmacodynamic evaluation of rocuronium included 80 surgical dogs but focused on dose-dependent neuromuscular block and did not compare ventilation or reversal strategies [29]. More recently, rocuronium–sugammadex was used to provide controlled apnea during stereotactic radiation therapy in 14 dogs undergoing 43 treatment sessions, with rapid reversal after treatment [30]. Larger canine anesthesia or ventilation cohorts evaluated transient postoperative hypoxemia in 654 dogs, dynamic respiratory-system compliance in 515 dogs, and outcomes of long-term mechanical ventilation in 227 dogs [12,31,32]. However, the first two studies did not compare NMBA selection, quantitative neuromuscular recovery, or reversal strategies, whereas the third examined long-term critical-care ventilation outside general anesthesia and included only 28 NMBA-exposed dogs. To our knowledge, the present 288-dog cohort—including 173 dogs managed with quantitatively monitored aminosteroidal neuromuscular blockade and pharmacologic reversal—is therefore the largest reported comparative perioperative cohort of client-owned dogs to jointly evaluate the ventilation strategy, aminosteroidal NMBA selection, quantitative neuromuscular monitoring and recovery, reversal strategy, primary-window physiology, emergence, and short-term postoperative outcomes. Its main contribution is therefore not simply another estimate of reversal speed, but a clinically interpretable contrast sequence that asks whether controlled ventilation alone was sufficient, what changed when neuromuscular blockade supported that ventilation, whether the NMBA strategy mattered under planned sugammadex reversal, and how sugammadex and neostigmine–atropine reversal compared after vecuronium.

### 4.2. Primary-Window Physiology: Controlled Ventilation Alone vs. NMBA-Facilitated Ventilation

The primary endpoint assessed simultaneous MAP–EtCO_2_ target attainment during the prespecified physiologic window, allowing ventilatory control to be interpreted alongside arterial-pressure control. Although the MAP threshold was aligned with veterinary guidance [5,6,7], it is not an absolute boundary between adequate and inadequate organ perfusion. Human evidence concerning the depth and duration of hypotension supports considering the burden of physiologic disturbances [8,9], but the combined MAP–EtCO_2_ endpoint is study-defined and has not been validated against clinical outcomes in dogs. Greater target attainment therefore indicates better performance against the study-defined physiologic targets; it does not, by itself, establish improved tissue perfusion or clinical outcomes.

The B-versus-A comparison demonstrates why the joint endpoint added information beyond EtCO_2_ alone. Planned controlled ventilation without an NMBA was associated with a modest improvement in EtCO_2_ target time and a modest reduction in EtCO_2_ dysregulation burden. In the principal baseline-adjusted analysis, these respiratory gains did not translate into improved concurrent physiology: the MAP target time was lower in Group B, the hypotension burden was not clearly reduced, and the adjusted joint target-time percentages were nearly identical. Group B also had the highest time-weighted mean end-tidal isoflurane concentration, the highest mean fentanyl CRI rate, frequent ventilator-setting changes, and ventilator asynchrony in 73.2% of dogs. Experimental and clinical canine studies show that ventilation mode and tidal-volume selection can materially influence cardiopulmonary variables, but they do not directly reproduce PRVC ventilation without NMBA in geriatric surgical patients [10,11,13]. Differences in delivered airway pressures may also have contributed to the observed hemodynamic pattern. The common initial ventilator-setting ranges did not ensure equivalent pressure exposure during subsequent clinical titration, and the observed asynchrony and setting-change counts do not establish poorer respiratory-system compliance or higher PEEP requirements in Group B. The present pattern is therefore consistent with, but does not prove, a cycle in which patient–ventilator discordance prompted additional anesthetic titration and ventilator adjustment, with improved CO_2_ control offset by less favorable arterial-pressure control. Formal temporal mediation was not evaluated, and reverse causation remains possible because clinicians may have intensified ventilation or anesthesia in response to instability already present.

The C-versus-B contrast provides the clearest observational indication that simply activating a ventilator was not equivalent to consistently delivering an effective controlled-ventilation strategy. Both groups were assigned to planned controlled ventilation, and every dog achieved at least 80% controlled-ventilation exposure, yet Group C had a 27.3-percentage-point higher adjusted joint target-time percentage. The same contrast favored Group C for EtCO_2_ and MAP target time, both dysregulation burdens, ventilator-setting changes, unplanned ventilatory intervention, dopamine use, and fluid-bolus use. Ventilator asynchrony occurred in only 3.4% of Group C compared with 73.2% of Group B, and no Group C dog required an unplanned ventilatory intervention. Reduced asynchrony is partly expected because neuromuscular blockade suppresses spontaneous respiratory activity; it may facilitate controlled ventilation but does not, by itself, establish a clinical benefit. However, the C-versus-B contrast does not isolate a single pharmacologic component: it compares no NMBA with a strategy incorporating rocuronium, NMBA-guided management, and subsequent sugammadex reversal. Because reversal occurred after the primary window, sugammadex itself cannot explain the primary-window joint-target advantage.

Group C also had a lower time-weighted mean EtISO and a lower mean fentanyl CRI rate than each of the other four groups while maintaining more favorable MAP and EtCO_2_ control. These observations are clinically relevant as anesthetic-management measures, but they require careful interpretation. Neuromuscular blocking agents provide neither hypnosis nor analgesia and remove movement as a clinical response to stimulation; consequently, lower recorded EtISO and fentanyl CRI exposure in an NMBA-treated dog cannot by itself establish an equivalent anesthetic depth or antinociception [14,33]. Fentanyl infusion can substantially reduce isoflurane MAC in dogs, confirming that the two exposures are pharmacodynamically interdependent [34]. Nevertheless, the present fentanyl variable represented only the mean CRI rate, excluded bolus doses, and was not accompanied by validated pain or sedation scores. It should therefore not be described as total opioid consumption, opioid sparing, superior analgesia, or reduced pain. Lower isoflurane and fentanyl exposure may have contributed to the observed arterial-pressure profile through reduced cardiovascular depression, less suppression of sympathetic responses to surgical stimulation, or both. These mechanisms cannot be distinguished from the available data. Additional adjustment for mean EtISO and fentanyl CRI reduced the estimated differences but retained higher joint target-time percentages in Group C than in Groups A, B, and D. These findings support the association between the implemented Group C strategy and superior concurrent physiologic target attainment while emphasizing the relevance of accompanying anesthetic management.

The hemodynamic-support pattern reinforced, but did not independently prove, that interpretation. No Group C dog received dopamine, and only 2 of 59 received a qualifying crystalloid bolus, compared with 11 of 59 and 20 of 59 in Group A and 16 of 56 and 26 of 56 in Group B, respectively. These findings were directionally concordant with Group C’s higher MAP target time and lower hypotension burden. However, dopamine and the fluid bolus are process outcomes that depend on clinician recognition, treatment thresholds, and the response to preceding physiology. They should therefore be viewed as supportive markers of the management burden associated with each strategy, not independent confirmation of causal hemodynamic protection.

### 4.3. NMBA Choice, Quantitative Reversal, and Emergence

The C-versus-D contrast compared two planned controlled-ventilation strategies that both incorporated quantitative monitoring and subsequent sugammadex reversal but used different aminosteroidal NMBAs. Group C had a 12.7-percentage-point higher adjusted joint target-time percentage, higher EtCO_2_ and MAP target time, lower dysregulation burdens, lower time-weighted mean EtISO and mean fentanyl CRI rate, fewer ventilator-setting changes, and faster quantitative recovery and emergence. Recovery to normalized TOFR ≥ 0.90 and ≥1.00 and reversal-to-extubation time remained faster in Group C after additional adjustment for baseline TOFR, TOFC at reversal, temperature, residual EtISO, and the final-NMBA-to-reversal interval. These observations suggest a more favorable rocuronium-based strategy under the dosing and management conditions of this cohort. They do not establish an intrinsic or universal pharmacologic superiority of rocuronium over vecuronium. Earlier canine work established the clinical feasibility of rocuronium administered as bolus doses or continuous infusion, whereas Mosing et al. demonstrated rapid sugammadex reversal after profound blockade with either rocuronium or vecuronium [14,15,16,17]. Differences among the present groups may reflect agent-specific kinetics, repeated-dose behavior, block depth, dose timing, clinician titration, and the particular TOFC-guided sugammadex regimen. The C–D comparison is therefore best interpreted as a comparison of two implemented NMBA strategies rather than an equipotent, randomized drug comparison. Data comparing rocuronium with cisatracurium further demonstrate variability in duration and recovery but do not directly resolve the rocuronium–vecuronium contrast [35].

The D-versus-E comparison provides the most direct clinical contrast between reversal approaches after vecuronium. Adjusted mean recovery to normalized TOFR ≥ 0.90 was 2.31 min with sugammadex and 7.43 min with neostigmine–atropine; corresponding times to normalized TOFR ≥ 1.00 were 11.58 and 17.84 min, and reversal-to-extubation times were 9.45 and 12.61 min. The direction remained after restriction to TOFC 2–4 and additional adjustment for cumulative vecuronium dose and conditions at reversal. These findings align closely with a randomized canine clinical trial in which vecuronium recovery was dose-dependently faster with sugammadex than with neostigmine and more variable after neostigmine or lower-dose sugammadex [23]. They are also consistent with earlier canine neostigmine dose–response data and with human randomized and systematic-review evidence showing more rapid reversal with sugammadex [18,36,37,38].

The temporal boundary of this inference is crucial. In Groups C–E, reversal was administered after discontinuation of isoflurane, whereas the primary physiologic window ended at isoflurane discontinuation. Thus, the 6.1-percentage-point D-versus-E difference in primary joint target time preceded administration of either reversal agent and cannot be attributed causally to sugammadex rather than neostigmine–atropine. That pre-reversal difference may reflect baseline selection, management differences, or other residual confounding between two nonrandomized strategy groups. The reversal-agent contrast is supported specifically by post-administration quantitative recovery and emergence outcomes. Likewise, although postoperative event risks were numerically lower in Group D, no principal D-versus-E safety comparison remained statistically significant. The data therefore support faster attainment of the prespecified quantitative recovery thresholds and faster emergence with the sugammadex strategy, not proven superiority in postoperative clinical safety.

Quantitative monitoring was fundamental to interpreting these recovery data. Restoration of spontaneous ventilation does not exclude residual neuromuscular blockade in dogs [19], and quantitative recovery estimates can differ among monitoring technologies [24,33]. All 173 NMBA-treated dogs achieved the prespecified normalized TOFR ≥ 0.90 before extubation, and monitoring continued after extubation until the stricter supportive TOFR ≥ 1.00 endpoint was evaluated. This additional endpoint is especially pertinent because three-axis acceleromyography may overestimate neuromuscular recovery relative to mechanomyography in dogs; in a direct comparison, use of a ratio of 1.00 rather than 0.90 reduced, but did not eliminate, false-positive recovery classifications [27]. The NMS450X configuration used in the present cohort was consistent with published canine clinical application [22], while the baseline normalization, repeated threshold confirmation, and continued post-extubation monitoring strengthened interpretability. The finding that TOFR ≥ 1.00 was first documented after extubation in 57.6%, 64.3%, and 96.6% of Groups C, D, and E, respectively, shows that the stricter supportive threshold had not yet been documented before extubation in many dogs, particularly in Group E; it should not be equated with clinically apparent residual paralysis or unsafe extubation. Human consensus and professional guidelines similarly emphasize quantitative monitoring and recovery to TOFR ≥ 0.90 [25,26,39], whereas the Geneva revision provides methodological standards for pharmacodynamic studies of neuromuscular blocking agents [40]. These human thresholds and their outcome implications cannot be transferred uncritically to dogs or across monitoring technologies.

### 4.4. Peri-Anesthetic Safety and Integrated Clinical Interpretation

Pre-extubation safety findings were directionally coherent with the continuous physiologic outcomes but were not uniformly superior in Group C. Dopamine and fluid-bolus use were lowest in Group C, yet ECG-confirmed bradyarrhythmia and rescue anticholinergic use did not differ among strategies, and these two endpoints represented the same 15 dogs rather than independent events. Only two dogs received epinephrine. Pre-extubation hypoxemia differed in the omnibus comparison, but none of the five prespecified pairwise contrasts remained significant after multiplicity correction. Boundary analyses for the five unassessable oxygenation records preserved evidence of overall heterogeneity but did not produce a significant prespecified contrast. Consequently, the results do not support a claim that Group C was superior for every pre-extubation safety endpoint. They instead show that the superior joint physiology observed in Group C was accompanied by low observed use of hemodynamic support without an evident increase in bradyarrhythmia or hypoxemia.

The postoperative event pattern gave the primary physiologic association greater clinical coherence. Early respiratory events occurred in 3.4% of Group C, compared with 40.7% and 44.6% of Groups A and B, and hard events within 48 h occurred in 1.7%, compared with 18.6% and 17.9%, respectively. The C-versus-A and C-versus-B differences remained significant after Holm adjustment in the principal exact analyses. Nevertheless, only 28 hard events occurred, the hard-event estimates were observed and non-covariate-adjusted, and the event composites were study-defined rather than validated veterinary morbidity scales. The early, major, and hard windows also overlapped: all dogs with a hard event had an early respiratory event, and the three counts cannot be summed or interpreted as independent outcomes or sequential severity stages. The exploratory adjusted early-event model supported the same direction but produced out-of-range individual fitted probabilities and cannot replace the principal exact analysis. These postoperative safety findings should therefore be interpreted as descriptive and hypothesis-generating.

Residual neuromuscular blockade can impair upper-airway function and has been associated with adverse respiratory recovery in human studies [41,42,43,44,45]. In a randomized trial, intraoperative acceleromyographic monitoring reduced residual blockade and adverse respiratory events [46]. Large perioperative human studies, however, are not fully concordant. Some observational cohorts associated intermediate-acting NMBAs or reversal choice with postoperative pulmonary complications; STRONGER and STIL-STRONGER favored sugammadex over neostigmine, whereas POPULAR did not demonstrate that neuromuscular monitoring, reversal, or sugammadex use was associated with lower pulmonary-complication risk [47,48,49,50]. Randomized and meta-analytic data support more reliable recovery with sugammadex, but clinical pulmonary benefits remain context-dependent [38,51]. These human data provide biologic and clinical context rather than direct evidence for the magnitude or causality of effects in geriatric small dogs. Moreover, Groups A and B received no NMBA, so the lower event rate in Group C cannot be explained solely by prevention of residual blockade. Primary-window physiologic control, recorded time-weighted EtISO and fentanyl CRI exposure, patient–ventilator interaction, emergence, and postoperative monitoring may all have contributed, but the present data do not establish a mediating pathway.

Figure 10 therefore serves as a descriptive synthesis, not a formal benefit–risk analysis. Group C combined the highest adjusted joint target-time estimate with the lowest observed early and hard-event risks, indicating that its primary physiologic advantage was accompanied by, rather than traded against, low observed postoperative event risks. The C-versus-D early and hard-event differences were not significant in the principal exact analyses, although the exploratory adjusted early-event estimate favored Group C, and neither D-versus-E safety contrast was statistically supported. It is appropriate to describe Group C as showing the most favorable integrated descriptive profile across the displayed outcomes. It would not be appropriate to call Figure 10 a validated composite, a formal ranking, or proof that Group C was the safest strategy.

### 4.5. Strengths and Limitations

Several design features strengthen this study. The cohort comprised 288 unique geriatric dogs weighing ≤7 kg with similar numbers in each strategy group and complete 48 h follow-up. Data were obtained within a single center under a consistent surgical and anesthetic framework, limiting heterogeneity in equipment, monitoring, and routine management. The primary endpoint used dog-level duration-weighted physiology rather than treating repeated monitor observations as independent measurements, applied the entire primary-window denominator, and was supported by high paired coverage. Prespecified contrasts decomposed clinically relevant strategy questions, and multiplicity was controlled within defined inferential families. Quantitative baseline-normalized TOF monitoring, confirmation of recovery thresholds, and post-extubation monitoring were available throughout the NMBA-treated cohort. Finally, the principal effect was examined using complementary models, crossover and coverage restrictions, overlap weighting, duration adjustment, and bootstrap resampling, and case-level values and definitions were retained in Supplementary Table S1. These features provide unusually detailed physiologic and recovery characterization for a veterinary retrospective anesthesia cohort.

The principal limitation is the nonrandomized, single-center retrospective design. Strategy selection was a clinical decision, creating the possibility of confounding by indication, and measured adjustment cannot account for unrecorded factors such as subtle differences in respiratory effort, anticipated surgical immobility, clinician concern, or evolving intraoperative conditions. Baseline imbalance was appreciable for age, cardiac status, ASA status, body weight, breed, and urgency. The breed was not included in the adjusted models because of sparse categories, leaving potential breed-related residual confounding, particularly for respiratory outcomes. Several baseline adjustment variables were based on contemporaneous clinician-recorded categories rather than externally validated classification algorithms, and some residual misclassification is therefore possible. Although the prespecified sensitivity analyses were directionally consistent, maximum absolute SMD after overlap weighting remained 0.40, indicating that weighting did not achieve ideal balance. The groups also represent complete clinical strategies rather than isolated interventions. Isoflurane and fentanyl exposures were titrated during the outcome window and could reflect both the selected strategy and responses to evolving physiology. Their strong correlation and limited between-group overlap made some drug-adjusted contrasts dependent on extrapolation and restricted attribution to individual strategy components. C-versus-D is not an equipotent randomized NMBA comparison, and the pre-reversal D-versus-E primary difference cannot be caused by the later reversal agent. All causal language must therefore remain bounded by the observational design.

Measurement and outcome limitations also warrant emphasis. Joint target time and both burden metrics were prespecified study-derived measures and have not been externally validated against canine organ injury, recovery quality, or mortality. MAP was assigned from intermittent noninvasive oscillometric measurements rather than continuous invasive arterial pressure, and EtCO_2_ is not interchangeable with arterial PaCO_2_. Patient-level measurements of delivered tidal volume, respiratory rate, respiratory-system compliance, and airway-pressure exposures (peak, plateau, and PEEP) were not included in the analytical dataset, limiting assessment of their contribution to the between-strategy associations. Although signal rules were fixed and coverage was high, minute-level summarization and invalid epochs may have reduced short-lived variability. Sensitivity to starting the primary window at tracheal intubation or the first valid paired MAP–EtCO_2_ measurement was not directly evaluated. Three-axis acceleromyography may overestimate recovery relative to mechanomyography, even with normalization and the supportive 1.00 threshold. The absence of total fentanyl bolus exposure, validated perioperative pain scores, sedation scores, and objective anesthetic-depth measures limits interpretation of the lower time-weighted mean EtISO and fentanyl CRI rates. Dopamine, fluid bolus, ventilator adjustment, and escalation events combined physiology with clinician behavior and were not blinded outcomes. Event extraction was performed retrospectively, and several composites were study-defined and overlapping.

Finally, safety events were sparse relative to the number of covariates and contrasts. This precluded stable covariate-adjusted modeling of the hard and major-event endpoints and resulted in wide exact confidence intervals for several group comparisons. Modified-Poisson analyses of fluid-bolus use and early respiratory events were explicitly exploratory because some individual fitted probabilities exceeded 1, although the marginal standardized risks remained within the probability bounds. The corresponding asynchrony model was not interpreted because the boundary violations were accompanied by high leverage. The cohort was restricted to dogs aged at least 12 years and weighing no more than 7 kg, 91% underwent orthopedic surgery, and only 26 underwent thoracolumbar hemilaminectomy. The absence of detected interaction by surgical stratum or ASA category should not be interpreted as equivalence because subgroup power was limited. Use of one institution, a consistent clinical team, a specific PRVC platform, and a specific quantitative monitor improves internal consistency but restricts external generalizability to younger or larger dogs, thoracic or abdominal surgery, other ventilators, and less standardized settings. Follow-up was limited to 48 h and cannot address delayed morbidity or long-term functional recovery.

### 4.6. Clinical Implications and Future Research

The clinically relevant exposure in this study was a system-level strategy comprising planned controlled ventilation, reliable neuromuscular blockade, quantitative TOF monitoring, depth-appropriate reversal, and monitored emergence. The findings should not be simplified to a recommendation to administer rocuronium or sugammadex in isolation, and NMBA use without adequate hypnosis, analgesia, ventilatory capability, and quantitative monitoring would not reproduce the evaluated strategy. Implementation of these strategies in other clinical settings may also depend on drug availability, clinician familiarity, and cost. Current veterinary monitoring guidance and reviews support quantitative assessment and protocolized reversal when NMBAs are used [6,7,14,33], and recent canine clinical experience illustrates the expanding implementation of rocuronium–sugammadex strategies [30]. In geriatric small dogs requiring immobility and controlled ventilation, the present results support prospective evaluation of the complete Group C strategy. A multicenter randomized or pragmatic comparative trial should preserve the temporal distinction between the primary physiologic window and post-isoflurane reversal, include invasive or more frequent arterial-pressure assessment and arterial blood gases where feasible, record total opioid exposure and validated pain and sedation scores, prespecify patient-centered recovery outcomes, and extend follow-up beyond 48 h. Such a design is required to determine whether the robust physiologic associations observed here represent causal improvements in clinical safety and recovery.

## 5. Conclusions

In this retrospective cohort of 288 geriatric small dogs, dogs managed with the rocuronium-facilitated controlled-ventilation strategy showed superior concurrent MAP–EtCO_2_ target attainment compared with those managed with spontaneous ventilation or controlled ventilation without neuromuscular blockade. These observational findings concern a study-defined physiologic endpoint that has not been validated against clinical outcomes in dogs; prospective studies are needed to determine whether the observed physiologic advantage translates into a clinical benefit.

## Figures and Tables

**Table 1:** Baseline demographic, preanesthetic, and procedural characteristics of the 288 dogs according to initially planned anesthetic strategy.

Characteristic	Overall (*n* = 288)	Group A (*n* = 59)	Group B (*n* = 56)	Group C (*n* = 59)	Group D (*n* = 56)	Group E (*n* = 58)	Maximum |SMD| (Pair)
Age, months, mean (SD)	167.2 (14.2)	165.6 (14.3)	168.2 (13.4)	176.6 (14.1)	164.4 (11.6)	161.0 (12.9)	1.160 (C–E)
Body weight, kg, mean (SD)	4.21 (1.30)	4.35 (1.22)	3.85 (1.25)	4.21 (1.37)	4.26 (1.33)	4.40 (1.31)	0.430 (B–E)
**Sex/neuter status, *n* (%)** Female intact	12 (4.2)	3 (5.1)	2 (3.6)	2 (3.4)	3 (5.4)	2 (3.4)	0.387 (B–C)
Female spayed	131 (45.5)	25 (42.4)	22 (39.3)	34 (57.6)	22 (39.3)	28 (48.3)	
Male intact	13 (4.5)	3 (5.1)	3 (5.4)	3 (5.1)	3 (5.4)	1 (1.7)	
Male neutered	132 (45.8)	28 (47.5)	29 (51.8)	20 (33.9)	28 (50.0)	27 (46.6)	
**Breed, *n* (%)** Bichon Frise	25 (8.7)	3 (5.1)	4 (7.1)	5 (8.5)	5 (8.9)	8 (13.8)	0.485 (A–E)
Chihuahua	29 (10.1)	5 (8.5)	6 (10.7)	6 (10.2)	6 (10.7)	6 (10.3)	
Maltese	42 (14.6)	9 (15.3)	8 (14.3)	10 (16.9)	8 (14.3)	7 (12.1)	
Mixed	21 (7.3)	3 (5.1)	4 (7.1)	6 (10.2)	3 (5.4)	5 (8.6)	
Pomeranian	87 (30.2)	17 (28.8)	21 (37.5)	19 (32.2)	15 (26.8)	15 (25.9)	
Poodle	35 (12.2)	10 (16.9)	5 (8.9)	8 (13.6)	7 (12.5)	5 (8.6)	
Shih Tzu	31 (10.8)	9 (15.3)	5 (8.9)	4 (6.8)	7 (12.5)	6 (10.3)	
Spitz	18 (6.2)	3 (5.1)	3 (5.4)	1 (1.7)	5 (8.9)	6 (10.3)	
**ASA physical status, *n* (%)** II	108 (37.5)	20 (33.9)	29 (51.8)	19 (32.2)	19 (33.9)	21 (36.2)	0.480 (B–D)
III	138 (47.9)	29 (49.2)	20 (35.7)	28 (47.5)	33 (58.9)	28 (48.3)	
IV	42 (14.6)	10 (16.9)	7 (12.5)	12 (20.3)	4 (7.1)	9 (15.5)	
**Cardiac status, *n* (%)** None	111 (38.5)	21 (35.6)	27 (48.2)	16 (27.1)	20 (35.7)	27 (46.6)	0.523 (B–C)
Subclinical	92 (31.9)	24 (40.7)	17 (30.4)	18 (30.5)	17 (30.4)	16 (27.6)	
Clinically significant	59 (20.5)	8 (13.6)	8 (14.3)	16 (27.1)	16 (28.6)	11 (19.0)	
CHF history	26 (9.0)	6 (10.2)	4 (7.1)	9 (15.3)	3 (5.4)	4 (6.9)	
**Renal or respiratory risk, *n* (%)** None	163 (56.6)	34 (57.6)	34 (60.7)	33 (55.9)	31 (55.4)	31 (53.4)	0.301 (C–D)
Renal only	54 (18.8)	13 (22.0)	7 (12.5)	9 (15.3)	12 (21.4)	13 (22.4)	
Respiratory only	50 (17.4)	8 (13.6)	10 (17.9)	14 (23.7)	8 (14.3)	10 (17.2)	
Both	21 (7.3)	4 (6.8)	5 (8.9)	3 (5.1)	5 (8.9)	4 (6.9)	
**Urgency, *n* (%)** Elective	166 (57.6)	36 (61.0)	28 (50.0)	38 (64.4)	32 (57.1)	32 (55.2)	0.435 (B–E)
Urgent	91 (31.6)	18 (30.5)	16 (28.6)	16 (27.1)	19 (33.9)	22 (37.9)	
Emergency	31 (10.8)	5 (8.5)	12 (21.4)	5 (8.5)	5 (8.9)	4 (6.9)	
**Surgical stratum, *n* (%)** Orthopedic	262 (91.0)	54 (91.5)	54 (96.4)	52 (88.1)	50 (89.3)	52 (89.7)	0.315 (B–C)
Thoracolumbar IVDE	26 (9.0)	5 (8.5)	2 (3.6)	7 (11.9)	6 (10.7)	6 (10.3)	
**Procedural complexity, *n* (%)** S1	89 (30.9)	19 (32.2)	17 (30.4)	16 (27.1)	19 (33.9)	18 (31.0)	0.361 (C–E)
S2	108 (37.5)	22 (37.3)	23 (41.1)	26 (44.1)	21 (37.5)	16 (27.6)	
S3	91 (31.6)	18 (30.5)	16 (28.6)	17 (28.8)	16 (28.6)	24 (41.4)	
**Anesthetist, *n* (%)** AN01	150 (52.1)	31 (52.5)	29 (51.8)	31 (52.5)	29 (51.8)	30 (51.7)	0.016 (A–E)
AN02	138 (47.9)	28 (47.5)	27 (48.2)	28 (47.5)	27 (48.2)	28 (48.3)	

Values are mean (SD) or *n* (%). Percentages are calculated within columns. No null-hypothesis significance tests were performed for baseline characteristics. The reported SMD is the largest absolute value among the ten pairwise strategy-group comparisons. For continuous variables, the SMD was the difference in group means divided by the square root of the average of the two group variances. For multilevel categorical variables, a generalized multinomial Mahalanobis SMD was used. ASA, American Society of Anesthesiologists; CHF, congestive heart failure; IVDE, intervertebral disc extrusion; SD, standard deviation; SMD, standardized mean difference.

**Table 2:** Primary joint target-time outcome and prespecified strategy contrasts.

Part A. Strategy-specific estimates.					
**Strategy**	***n***	**Observed Mean (SD), %**		**Adjusted Marginal Mean (95% CI), %**	
Group A	59	59.2 (4.2)		58.9 (58.3–59.5)	
Group B	56	59.4 (4.4)		59.3 (58.6–60.0)	
Group C	59	84.4 (3.2)		86.6 (85.7–87.5)	
Group D	56	74.6 (3.8)		74.0 (73.3–74.7)	
Group E	58	69.0 (4.4)		67.8 (67.1–68.6)	
Part B. Five prespecified primary contrasts					
**Contrast**	**Unadjusted Difference (Simultaneous 95% CI), pp**		**Adjusted Difference (Simultaneous 95% CI), pp**		**Single-Step Adjusted *p***
C versus A	25.2 (23.5–27.0)		27.7 (26.3–29.2)		<0.001
B versus A	0.2 (−1.8–2.3)		0.4 (−0.8–1.6)		0.876
C versus B	25.0 (23.2–26.8)		27.3 (25.9–28.8)		<0.001
C versus D	9.8 (8.1–11.5)		12.7 (11.2–14.1)		<0.001
D versus E	5.6 (3.6–7.5)		6.1 (4.9–7.3)		<0.001

Overall adjusted strategy-group effect: HC3-robust Wald χ^2^ = 3394.97, df = 4, *p* < 0.001. Values are observed means (SDs) or model-based estimates with 95% CIs. Contrasts were calculated from full-precision model estimates; consequently, they may not equal differences obtained from the displayed rounded marginal means. The prespecified adjustment set comprised age, body weight, sex/neuter status, ASA physical status, cardiac status, renal or respiratory risk, urgency, surgical stratum, procedural complexity, and anesthetist code. Contrasts are first-named minus second-named group. Simultaneous CIs and adjusted *p* values were obtained using the single-step procedure over the five prespecified contrasts. ASA, American Society of Anesthesiologists; CI, confidence interval; pp, percentage points; SD, standard deviation.

## Data Availability

The complete de-identified individual-level analysis dataset and accompanying data dictionary are available in Supplementary Table S1.

## Supplementary Materials

The following supporting information can be downloaded at: https://www.mdpi.com/article/10.3390/ani16203203/s1, Supplementary Table S1: De-identified individual-level analysis dataset for the 288 included dogs and accompanying data dictionary.
