# Supplementary material for: Superior MAP–EtCO2 Target Attainment with Rocuronium-Facilitated Controlled Ventilation in Geriatric Small Dogs: A Retrospective Cohort Study
Source: Animals (Basel). 2026 Oct 12;16(20):3203. doi: 10.3390/ani16203203 (PMC13700846; doi:10.3390/ani16203203)
Supplement: Supplementary file 1 [file animals-16-03203-s001.zip › Owner_Consent_Form.pdf]

# Owner Consent for Retrospective Use and Publication of De-identified Clinical Data

| | | | | |
|---|---|---|---|---|
| **Date of Consent** | 20____ . ____ . ____ | | | |
| **Study Title** | Retrospective Evaluation of Five Peri-Anesthetic Ventilation, Neuromuscular-Blockade, and Reversal Strategies in Geriatric Small Dogs | | | |
| **Principal Investigator** | Name | Seung-Hyun Kim | Affiliation | KSH Surgical Animal Medical Center, Gwangju, Republic of Korea |

## 1. Invitation and Voluntary Authorization

You are being asked to authorize the use of information already recorded during your dog's routine veterinary care for a retrospective research study. Please read this form carefully and ask the study veterinarian any questions you may have before deciding.

Your decision is entirely voluntary. If your questions have been answered to your satisfaction and you agree to the use and publication of your dog's de-identified clinical information as described below, please sign and date this form using the actual date of consent.

## 2. Nature of the Study

This is a retrospective, observational medical-record study. All surgical, anesthetic, ventilatory, neuromuscular-blocking, reversal, monitoring, and postoperative-care decisions were made for clinical reasons before the research analysis. The study introduced no random assignment, experimental intervention, or deviation from routine clinical care.

## 3. Purpose and Background of the Study

This study reviews medical records for client-owned dogs that were at least 12 years old, weighed no more than 7 kg, and underwent pelvic-limb orthopedic surgery or thoracolumbar hemilaminectomy for intervertebral disc extrusion.

It compares five routinely used peri-anesthetic strategies: spontaneous ventilation without a muscle-relaxing drug; controlled mechanical ventilation without a muscle-relaxing drug; rocuronium with controlled ventilation and sugammadex reversal; vecuronium with controlled ventilation and sugammadex reversal; and vecuronium with controlled ventilation and neostigmine–atropine reversal. All treatment decisions were made according to clinical judgment and were not assigned for research purposes.

The study evaluates concurrent blood-pressure and exhaled-carbon-dioxide target attainment, recorded anesthetic exposure and ventilatory management, quantitative neuromuscular recovery, emergence from anesthesia, and predefined peri-anesthetic events. These comparisons may improve knowledge of peri-anesthetic management in similar dogs, but participation provides no guaranteed direct benefit to your dog.

## 4. Clinical Records and Information to Be Reviewed

Authorized study personnel may review information already recorded during routine care, including age, weight, sex, breed, preanesthetic health classifications, diagnosis and procedure details, anesthetic and analgesic medications, ventilation and physiologic monitoring data, neuromuscular-blocking and reversal records, quantitative train-of-four measurements, supportive treatments, recovery and extubation information, and recorded events through 48 hours after extubation.

Only information generated during routine clinical care will be used. No additional examination, test, sample collection, medication, anesthesia, ventilatory intervention, monitoring procedure, hospital visit, or charge is required for this research, and no aspect of your dog's care was or will be changed for research purposes.

## 5. Risks, Benefits, Costs, and Protection of Information

This records-only study creates no additional physical risk to your dog. The principal foreseeable risk is an unintended loss of privacy. There is no guaranteed direct medical benefit to you or your dog, no payment for authorization, and no additional research-related cost. The findings may improve scientific understanding of peri-anesthetic care for similar dogs in the future.

## 6. Voluntary Authorization and Withdrawal

Authorization is voluntary. You may decline without giving a reason. Declining or withdrawing authorization will not affect your dog's current or future veterinary care, fees, access to services, or relationship with KSH Surgical Animal Medical Center.

Direct identifiers will be removed from the research dataset and replaced with a study code. Owner and animal names, chart numbers, contact details, exact dates, identifiable images, and free-text clinical notes will not be included in publications or the shared dataset. Signed forms and any temporary linkage information will be stored separately and accessed only by authorized personnel.

Study results may be presented at scientific meetings and published in print, electronic, online, open-access, translated, or reprinted formats. A de-identified individual-level dataset and data dictionary may be published online as supplementary research material and may be downloaded, shared, copied, and reused worldwide. Although direct identifiers will be removed and identifying detail minimized, a small residual risk of indirect identification cannot be completely eliminated, and the research team cannot fully control copies downloaded after public release.

You may request withdrawal by contacting the Principal Investigator before the de-identified dataset is finalized for public release. If you withdraw before that point and the record remains linkable, your dog's record will be removed from the research dataset and from analyses that have not yet been published. After data have been incorporated into published results or publicly released supplementary material, removal may no longer be possible, and published or downloaded copies cannot be recalled. Withdrawal will not affect your dog's care.

Questions about the study, privacy, or withdrawal may be directed to Seung-Hyun Kim, KSH Surgical Animal Medical Center (e-mail: trazet08@gmail.com).

## 7. Owner Authorization for Use and Publication of Clinical Data

I confirm that I am the legal owner of the dog named below or am authorized to give permission on the owner's behalf. I have read this form, received an explanation in language I understand, and had the opportunity to ask questions.

I understand that this is a retrospective review of existing clinical records; no anesthetic, ventilation, neuromuscular-blocking, reversal, monitoring, or other clinical intervention was assigned to my dog for research purposes.

I understand the privacy safeguards and residual risk, the limits on withdrawal after public release, and that de-identified individual-level data may be published online. I voluntarily authorize the research team to use, analyze, present, publish, and share my dog's de-identified clinical information for the study described in this form.

---

**SIGNATURE PAGE — OWNER AUTHORIZATION**

Study: Retrospective Evaluation of Five Peri-Anesthetic Ventilation, Neuromuscular-Blockade, and Reversal Strategies in Geriatric Small Dogs

Dog's name: ______________________________________________

Hospital chart number: ______________________________________ (institutional linkage only; not published)

Owner or authorized representative: ______________________________

Relationship/authority if not the owner: ___________________________

Signature: ___________________________________ Date: ____________________

---

Person obtaining consent: _______________________________________

Signature: ___________________________________ Date: ____________________

Principal Investigator: Seung-Hyun Kim

Signature: ___________________________________ Date: ____________________
